# Passive and semi-active heave compensator: Project design methodology and control strategies

**DOI:** 10.1371/journal.pone.0183140

**Published:** 2017-08-16

**Authors:** William Humberto Cuellar Sanchez, Tássio Melo Linhares, André Benine Neto, Eugênio Libório Feitosa Fortaleza

**Affiliations:** 1 Department of Mechanical Engineering/University of Brasília, UnB, Brasília, DF, Brazil; 2 Laboratory IMS, UMR 5218 CNRS, University of Bordeaux, 33405 Talence, France; Massachusetts Institute of Technology, UNITED STATES

## Abstract

Heave compensator is a system that mitigates transmission of heave movement from vessels to the equipment in the vessel. In drilling industry, a heave compensator enables drilling in offshore environments. Heave compensator attenuates movement transmitted from the vessel to the drill string and drill bit ensuring security and efficiency of the offshore drilling process. Common types of heave compensators are passive, active and semi-active compensators. This article presents 4 main points. First, a bulk modulus analysis obtains a simple condition to determine if the bulk modulus can be neglected in the design of hydropneumatic passive heave compensator. Second, the methodology to design passive heave compensators with the desired frequency response. Third, four control methodologies for semi-active heave compensator are tested and compared numerically. Lastly, we show experimental results obtained from a prototype with the methodology developed to design passive heave compensator.

## Introduction

Ocean waves cause the raising and sinking of floating offshore platforms. This motion affects offshore drilling process, causing drill bit and drill string damage, collapse of borehole wall and well kick. Heave compensator is a device used to compensate motion of heave platform and avoid its negative effects. In field applications, compensators are subjected to significant sprung mass variations. It ranges from 150*tones*, at 2*km* depth to 350*tones*, at 8*km* from start to finish of drilling.

The four categories of heave compensators; Passive Heave Compensator (PHC), Semi-Active Heave Compensator (SAHC), Active Heave Compensator (AHC) and Hybrid Heave compensator (HHC).

**Fig 1:**
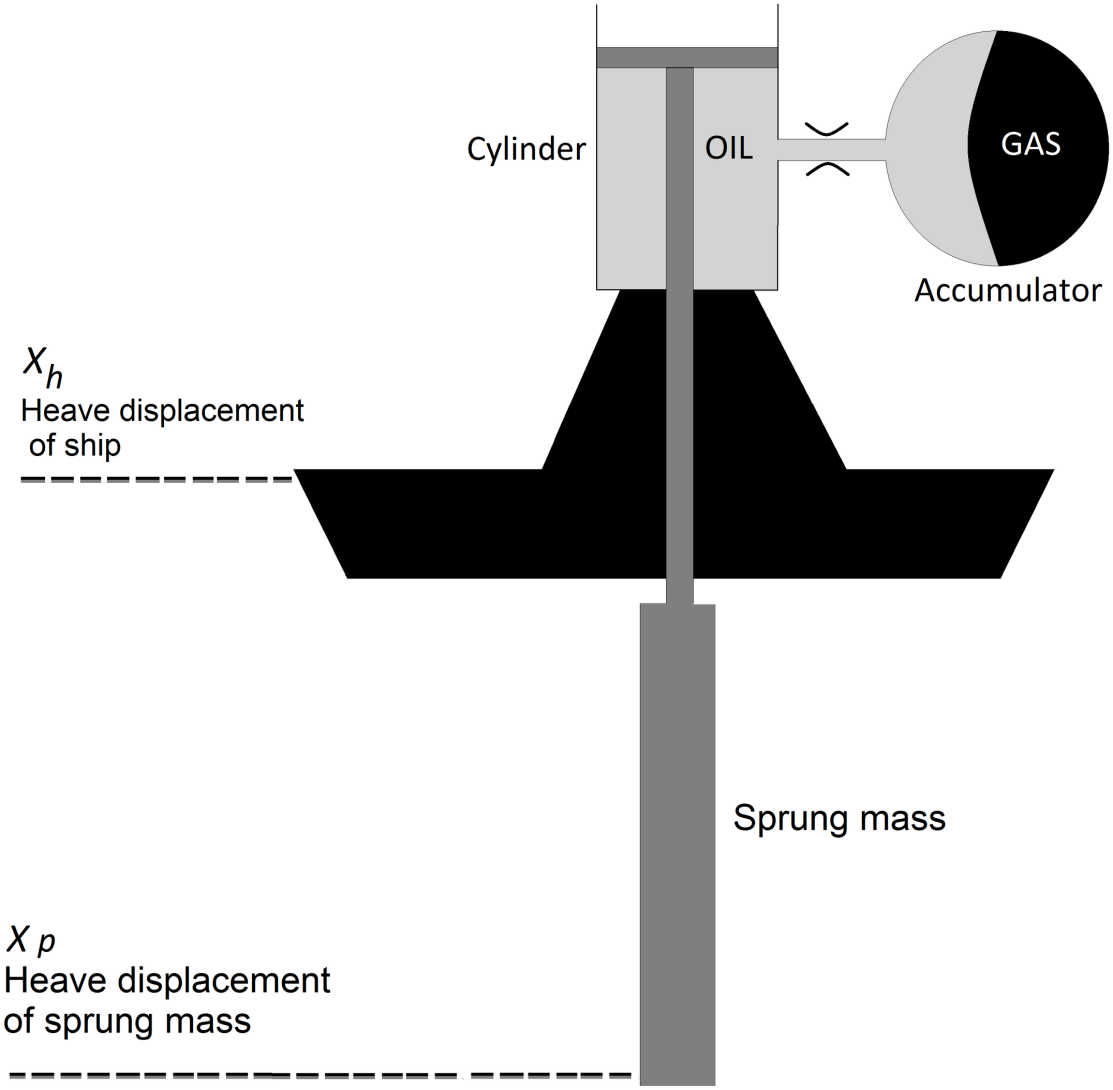
Diagram of traditional hydropneumatic suspension system.

PHCs are commonly hydropneumatic systems [1], consisting of gas spring and hydraulic damper, as illustrated in Fig 1. When modeling PHC, oil compressibility may be considered. In this case, the bulk modulus appears on the system equations. Considering bulk modulus, adding another differential equation to the model [2] increases complexity with no significant impact on model performance. Therefore, the requirements for the bulk modulus neglect are established. This reduces the PHC model and the complexity of its design, which can further be used in the design of controllers for HHC or SAHC applications.

Neglecting the bulk modulus, the relation between the physical parameters and the performance is obtained to design PHC. In this article, a methodology to design a PHC with the desired frequency response is proposed. For this purpose, a dimensionless factor is introduced to calculate the parameters of PHC based on performance specifications (cutoff frequency and supported mass). This methodology is used to design a reduced scale prototype, for experimental test and validation.

PHC have no external power source and utilizes the motion of system to develop control forces, however, the forces are not adjustable and cannot be controlled. Thus the attenuation rate (amplitude ratio between the output and input signal) is lower than 85% ([3] and [4]). On the other hand, AHCs are able to provide higher attenuation rates, above 95%. Which requires high energy consumption [5]. The AHC forces are adjustable. Sensors monitor the system and signals which are used to compute the control force [6] and [7].

HHC is obtained by the combination of an AHC and a PHC or a SAHC and a PHC [1, 8], for instance, [9] projected an HHC, where the passive holds 500*tonnes*, and the active requirement is 50*tonnes*. SAHC requires little external energy for operation and utilizes the motion of its own controlled system to develop the control forces, [8, 10–13]. This type of suspension is well known in automobile [3] and civil engineering fields [8] and [14]. Semi-active magnetorheological actuators are used in these fields, theirs controllers are designed considering an unknown disturbance, uncertain parameters and actuator limitations [15] and [16]. Examples of SAHC in the offshore drilling can be found in [13] and [17].

Change in mass affects the performance of PHC. Even though the AHC has high attenuation, it requires high energy consumption. For these reasons, two semi-active controllers are proposed. The first one modifies the damping as a function of sprung mass while the other modifies damping as a function of the sprung mass and its displacement, which is a kind of continuous balance control [18]. Servo-valve serves as an actuator control to modify damping.

The remainder of this paper is organized as follows; section Governing equations presents bulk modulus definition, the heave compensator model with and without consideration of the bulk modulus, and the condition to determine whether the bulk modulus can be neglected, thus obtaining a simplified model.

Section Dimensionless Factor introduces a dimensionless parameter and methodology to directly design the compensator with a desired performance. In section Semi-Active Heave Compensator (SAHC), semi-active controllers are proposed to maintain the desired frequency response. In section Experimental Validation, the experimental result shows the existence of dimensionless factor and the feasibility of the methodology to design PHC. Finally, section Conclusion summarizes the work.

## Governing equations

### Bulk modulus

Fluids have a degree of compressibility. Bulk modulus of a fluid is the inverse of its compressibility and represents the resistance of a fluid to compression. The bulk modulus is an important and inherent fluid property, because it indicates how fluid volume changes as external pressure is applied. Secant and the tangent bulk are two relevant expressions. The secant bulk expression is:

$$\begin{array}{c} \beta_{sec}=-V_{o}\frac{\Delta P}{\Delta V} \end{array}$$ (1)

where *β* is the bulk modulus, *V*_*o*_ is the initial volume, Δ*P* is the pressure variation and Δ*V* is the volume variation, this bulk modulus is more suitable for big pressure changes, because it represents an average or linear behaviour.

The tangent bulk modulus is more applicable for small dynamic pressure changes, it is also known as dynamic bulk modulus, which is expressed as:

$$\begin{array}{c} \beta_{tan}=-V\left(t\right)\frac{\partial P(t)}{\partial V} \end{array}$$ (2)

where *dP*/*dV* is the derivative of fluid pressure with respect to the nominal volume regarding the sprung mass, *V*(*t*) is the instantaneous volume of fluid during the compression. The tangent and secant bulk modulus could be isothermic or adiabatic, depending on the rate of pressure change.

Bulk modulus is not only a function of pressure, it is also affected by the type of hydraulic oil, oil temperature, air content in the oil and interface conditions between oil and air. When bulk modulus includes these effects, it is called the effective bulk modulus.

There are many models to describe bulk modulus for hydraulic fluids. For example, [19] simulated two models 1.7GPa and 0.3GPa.

### Heave compensator models

Under certain conditions, the bulk modulus can be neglected in a PHC mathematical model, simplifying the model. The simplified model is described by a simple transfer function of two poles and one zero, while the model with bulk modulus is described by three differential equations (associated to a transfer function of three poles and two zeros). The performance of the second order function is broadly similar to the more detailed non-linear heave compensator model [20]. These models depend on the sprung mass, which is not constant in the drilling process. It is essential to point out that these models are for non-contact operations.

### Heave compensator models with bulk modulus

According to [2], the assumptions below are made:

- The compensator works for non-contact operation.
- The hydraulic oil is compressible with the bulk modulus *K*, meaning the cylinder pressure variation affects the oil volume *V*_*E*_(*t*). The cylinder areas of rod chamber and area of non-rod chamber are considered equal to *A*.
- The sprung mass *M* varies in function of drill string length and it is considered constant for each depth.
- Only the heave displacement of sprung mass *x*_*p*_(*t*) and ship *x*_*h*_(*t*) are considered (see Fig 1).
- The cylinder has a linear damping force and its viscosity damping coefficient is *c*.
- When the compensator is absorbing the shocks during its regular operation, the compression of the gas takes place rapidly (between 0.01Hz and 1.5Hz, frequency of ocean waves), this compression is neither strictly isothermal nor purely adiabatic, it is a mixture of the two with a polytropic coefficient *r* of 1.33 as used in [21].
- The volume variations of oil in the accumulator are small compared to the total gas volume of the accumulator, allowing to linearize the gas state equation, establishing the variation of gas pressure as a function of flow of oil in the accumulator.
- The pipeline between the cylinder and the accumulator has a hydraulic conductivity *C*_*qR*_, which indicates the caracheteristic of the pipeline to transmit oil when it is submitted to a pressure gradient. Eq (3) is demonstraded in [2]:
  $$\begin{array}{c} C_{qR}=\frac{\pi d_{g}^{4}}{128\mu \iota } \end{array}$$ (3)
  where *d*_*g*_ is the inside diameter of the pipeline, *μ* is the dynamic viscosity of hydraulic oil and *ι* is the length of pipeline.

The PHC model proposed in [2] meets these assumptions:

$$\begin{array}{ccc} {\ddot{x}}_{p}\left(t\right) & = & -\frac{c}{M}{\dot{x}}_{p}\left(t\right)+\frac{A_{2}}{M}\Delta p_{E}\left(t\right)+\frac{c}{M}{\dot{x}}_{h}\left(t\right) \end{array}$$ (4)

$$\begin{array}{ccc} \Delta {\dot{p}}_{E}\left(t\right) & = & -\frac{KA_{2}}{V_{E}}{\dot{x}}_{p}\left(t\right)-\frac{KC_{qR}}{V_{E}}\Delta p_{E}\left(t\right)+\frac{KC_{qR}}{V_{E}}\Delta p_{G}\left(t\right)+\frac{KA_{2}}{V_{E}}{\dot{x}}_{h}\left(t\right) \end{array}$$ (5)

$$\begin{array}{ccc} \Delta {\dot{p}}_{G}\left(t\right) & = & \frac{rP_{G0}C_{qR}}{V_{G0}}\Delta p_{E}\left(t\right)-\frac{rP_{G0}C_{qR}}{V_{G0}}\Delta p_{G}\left(t\right) \end{array}$$ (6)

The static parameters at the operating point are: the accumulator gas volume *V*_*G*0_, the accumulator gas pressure *P*_*G*0_, the cylinder oil pressure *P*_*E*0_. The dynamic variables are *p*_*E*_(*t*) and *p*_*G*_(*t*) correspond respectively to the pressures of gas in accumulator and oil in cylinder respectively. Therefore, the small pressure variations Δ*p*_*E*_ and Δ*p*_*G*_ about equilibrium point can be defined as:

$$\begin{array}{ccc} \Delta p_{E}\left(t\right) & = & p_{E}\left(t\right)-P_{E0} \end{array}$$ (7)

$$\begin{array}{ccc} \Delta p_{G}\left(t\right) & = & p_{G}\left(t\right)-P_{G0} \end{array}$$ (8)

The expressions of static pressures are:

$$\begin{array}{c} \begin{array}{ccc} P_{E0} & = & \frac{Mg+P_{atm}A}{A}\\ P_{G0} & = & P_{E0} \end{array} \end{array}$$ (9)

### Heave compensator models without bulk modulus

Under the same assumptions as in the previous model, considering oil as incompressibile (i.e. infinity bulk modulus), Eq (4) is reduced to:

$$\begin{array}{c} \Delta p_{E}\left(t\right)=\Delta p_{G}\left(t\right)+\frac{A_{2}}{C_{qR}}\left({\dot{x}}_{h}\left(t\right)-{\dot{x}}_{p}\left(t\right)\right) \end{array}$$ (10)

Then the state equations are simplified:

$$\begin{array}{ccc} {\ddot{x}}_{p}\left(t\right) & = & -\frac{c}{M}{\dot{x}}_{p}\left(t\right)+\frac{A_{2}}{M}(\Delta p_{G}\left(t\right)+\frac{A_{2}}{C_{qR}}\left({\dot{x}}_{h}\left(t\right)-{\dot{x}}_{p}\left(t\right)\right))+\frac{c}{M}{\dot{x}}_{h}\left(t\right) \end{array}$$ (11)

$$\begin{array}{ccc} \Delta {\dot{p}}_{G}\left(t\right) & = & \frac{nA_{2}P_{G0}}{V_{G0}}\left({\dot{x}}_{h}\left(t\right)-{\dot{x}}_{p}\left(t\right)\right) \end{array}$$ (12)

This model can be represented in transfer function form as in [20], [17] and [1].

$$\begin{array}{c} \frac{x_{p}\left(s\right)}{x_{h}\left(s\right)}=\frac{\frac{c+b}{M}s+\frac{k}{M}}{s^{2}+\frac{c+b}{M}s+\frac{k}{M}} \end{array}$$ (13)

The relation between the transfer function and the state space is established with the expression of viscous damping coefficient *b* and the accumulator stiffness *k*, given by:

$$\begin{array}{c} b=A^{2}\frac{1}{C_{qR}}\;k=A^{2}r\frac{P_{G0}}{V_{G0}} \end{array}$$ (14)

## Condition to choose the model with or without bulk modulus

Simplification of the model is important to obtain simple controllers to facilitate the compensator design and performance analysis. The conditions to neglect the bulk modulus is described in this section. This conditions are obtained using the transmittance error expression, which is mathematically deduced from the concept of equivalent electrical impedances in S1 Appendix.

The oil is normally assumed to be incompressible in hydraulic applications. However, in hydropneumatic suspension systems, the bulk modulus needs to be taken into account at higher pressures. When gas is highly compressed and stiff spring is used, the bulk modulus should be added in the model to calculate the equivalent spring [22]. This means that, the gas stiffness dominates performance in low frequency range, while the oil stiffness exerts considerable influence over the transmissibility at higher frequencies ([23, 24]) and at high damping values.

Summarizing, the bulk modulus is important in some systems configuration, such as stiff stiffness, high pressures, high frequency disturbance and high damping. However, there is no literature indicating when bulk modulus is necessary in hydropneumatic suspension systems.

The influence of bulk modulus in the PHC is addressed with the frequency *s*_*b*_, which represents the maximum frequency value where the bulk modulus may be neglected, Then, when *s* < *s*_*b*_ the impedance and transmittances with and without bulk modulus are approximately the same (see S1 Appendix).

$$\begin{array}{c} s_{b}=\frac{1}{b}\sqrt{n^{2}{\left(\frac{KA^{2}}{V_{E}}\right)}^{2}-\left(\frac{rP_{G0}A^{2}}{V_{G0}}\right)} \end{array}$$ (15)

Therefore, when bulk modulus increases *s*_*b*_ also increases. This means an increase in frequency range where bulk modulus is neglected. Hydraulic suspensions show similar behavior for small values of bulk modulus [24]. The increase in oil volume also has a similar effect on the bulk modulus reduction (see [25] for an example on hydraulic systems).

The damping coefficient *b* is very relevant to the PHC performance, if this damping increases, the bulk modulus affects lower frequencies. A similar performance is shown in hydraulic systems. A suspension system with high value of *b* is studied in [26]. It presents an on–off switch-mode hydraulic circuit, when the system is in off mode, the fluid density increases storing energy in its compression. Therefore, the off mode of this system is similar to high value of damping coefficient *b*.

The condition is applied in the proposed PHC (details shown in section Numerical simulations), determining that the bulk modulus has no influence on the PHC performance.

## Dimensionless factor

The dimensionless factor *l* shows the relation between the natural frequency and the cutoff frequency. This factor has a value for each damping coefficient, which determines the maximum gain of frequency response of PHC (see subsection PHC design). This factor is used to design the PHC with the desired frequency response, the PHC parameters are expressed in function of the cutoff frequency and the factor *l*, which depends on damping ratio *ζ*. Thereafter, the equation to obtain the value of dimensionless factor in function of damping coefficient is acquired.

The relation between the cutoff frequency *ω*_*c*_ and the natural frequency *ω*_*n*_ is:

$$\begin{array}{c} \omega_{n}=l\left(\zeta \right)\omega_{c} \end{array}$$ (16)

The PHC from Eq (13) is a second order system with one zero.

$$\begin{array}{c} \frac{\left(\frac{b+c}{M}s+\frac{k}{M}\right)}{\left(s^{2}+\frac{b+c}{M}s+\frac{k}{M}\right)}=\frac{2\zeta \omega_{n}s+\omega_{n}^{2}}{\left(s^{2}+2\zeta \omega_{n}s+\omega_{n}^{2}\right)} \end{array}$$ (17)

The natural frequency and the damping coefficient are related to heave compensator. The natural frequency *ω*_*n*_ is substituted by Eq (16) and static pressure (*P*_*E*0_) by Eq (9) in the above relations, resulting in the following equations:

$$\begin{array}{c} b=2\zeta M\omega_{c}l-c\\ k={\left(\omega_{c}l\right)}^{2}M\\ V_{G0}=rA{\left(\frac{1}{l\omega_{c}}\right)}^{2}\frac{1}{M}\left(Mg+P_{atm}A\right) \end{array}$$ (18)

The compensator is designed with the desired cutoff frequency and damping. Now, the value of *l* in function of damping ratio is found. The transfer function Eq (17) is evaluated at cutoff frequency, the above equation is used, therefore *s* = *iω*_*n*_/*l*, simplifying *ω*_*n*_ yields:

$$\begin{array}{c} \frac{x_{p}\left(i\omega_{c}\right)}{x_{h}\left(i\omega_{c}\right)}=\frac{1+\frac{2\zeta }{l}i}{\left(1-\frac{1}{l^{2}}\right)-\frac{2\zeta }{l}i} \end{array}$$ (19)

The gain of Eq (19) is:

$${\left\|\frac{x_{p}\left(i\omega_{c}\right)}{x_{h}\left(i\omega_{c}\right)}\right\|}^{2}=\frac{1+\frac{4\zeta^{2}}{l^{2}}}{\frac{1}{l^{4}}+2\frac{1}{l^{2}}\left(2\zeta^{2}-1\right)+1}$$ (20)

The next equation is solved to find *l* in function of *ζ*.

$${\left\|\frac{x_{p}\left(i\omega_{c}\right)}{x_{h}\left(i\omega_{c}\right)}\right\|}^{2}+l^{2}\left(2\left(2\zeta^{2}-1\right){\left\|\frac{x_{p}\left(i\omega_{c}\right)}{x_{h}\left(i\omega_{c}\right)}\right\|}^{2}-4\zeta^{2}\right)+l^{4}\left({\left\|\frac{x_{p}\left(i\omega_{c}\right)}{x_{h}\left(i\omega_{c}\right)}\right\|}^{2}-1\right)=0$$ (21)

This dimensionless factor is used to design the compensator in section Numerical Simulations, showing that the PHC has the desired performance.

### PHC design

A simple process to design a heave compensator with a desired frequency response is shown here, using the model without bulk modulus.

**Fig 2:**
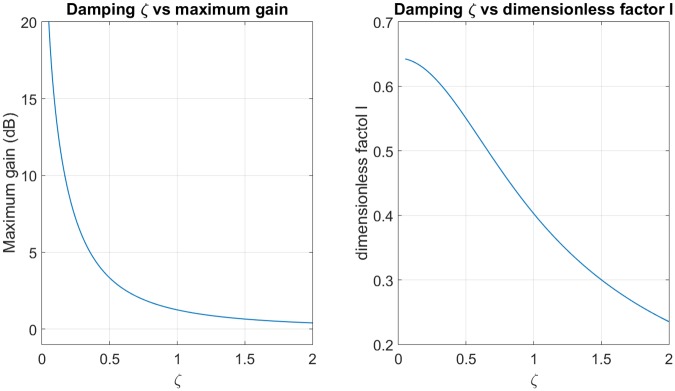
Parameter to project the compensator. (a) Maximum gain in function of damping coefficient. (b) Dimensionless factor in function of damping coefficient.

Firstly, a frequency response constraint is imposed. It defines the desired cutoff frequency *ω*_*c*_ (which usually has a gain of -3dB) and the maximum gain frequency in passband. Then the damping ratio *ζ* can be deduced from Fig 2A, which represents the maximum gain of frequency response in function of *ζ*. Using Eq (21) it is possible to relate the dimensionless factor *l* to the damping ratio *ζ* for a typical value of gain at *ω*_*c*_, as shown for instance in Fig 2B for −3*dB*.

Considering physical parameters, such as the maximum mass *m*_*max*_, the maximum pressure *P*_*max*_ and the cylinder damping coefficient *c*, it is possible to calculate the cylinder area using Eq (22). The cylinder area must be the minimum possible, in order to provide small accumulator volume. This volume is a critical point in the design of PHC, since the required volume is usually considerably large to ensure the desired performance ([17] and [2]). However, the cylinder area has minimum limit, because the accumulator pressure is inversely proportional to this parameter. The cylinder area is calculated with the maximum allowed pressure, *P*_*max*_.

$$\begin{array}{c} A=\frac{Mg}{P_{max}-P_{atm}} \end{array}$$ (22)

Finally, the compensator is designed by using the parameters *k*, *b*, *V*_*G*0_ from Eq (18) and the physical parameters related to the frequency response.

**Fig 3:**
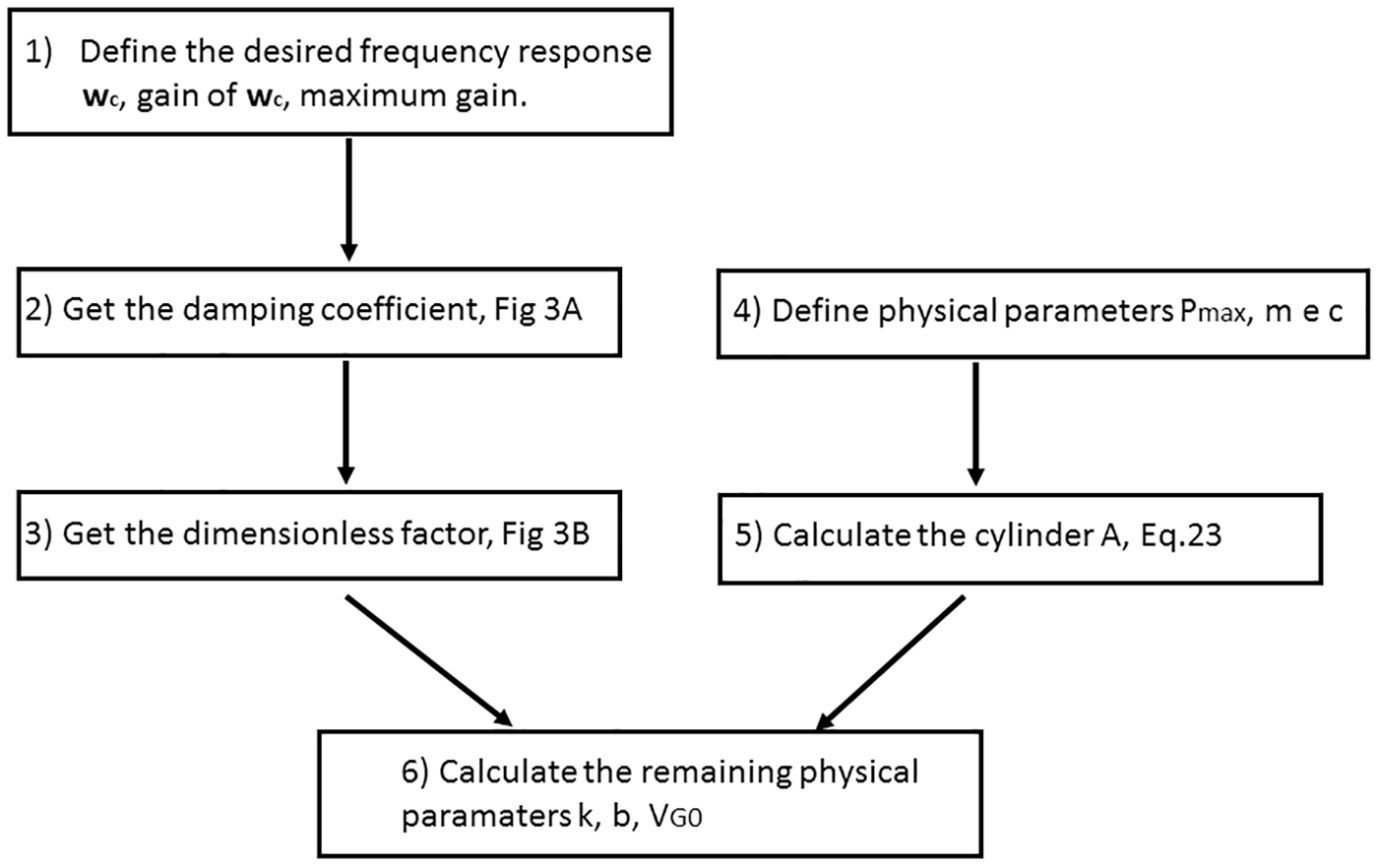
Diagram: Steps to project a hydropneumatic PHC.

This process ensures that the PHC has a required frequency performance (maximum gain and cutoff frequency) and its volume is minimum with an acceptable pressure value. The process is summarized in Fig 3.

## Semi-active heave compensator (SAHC)

**Fig 4:**
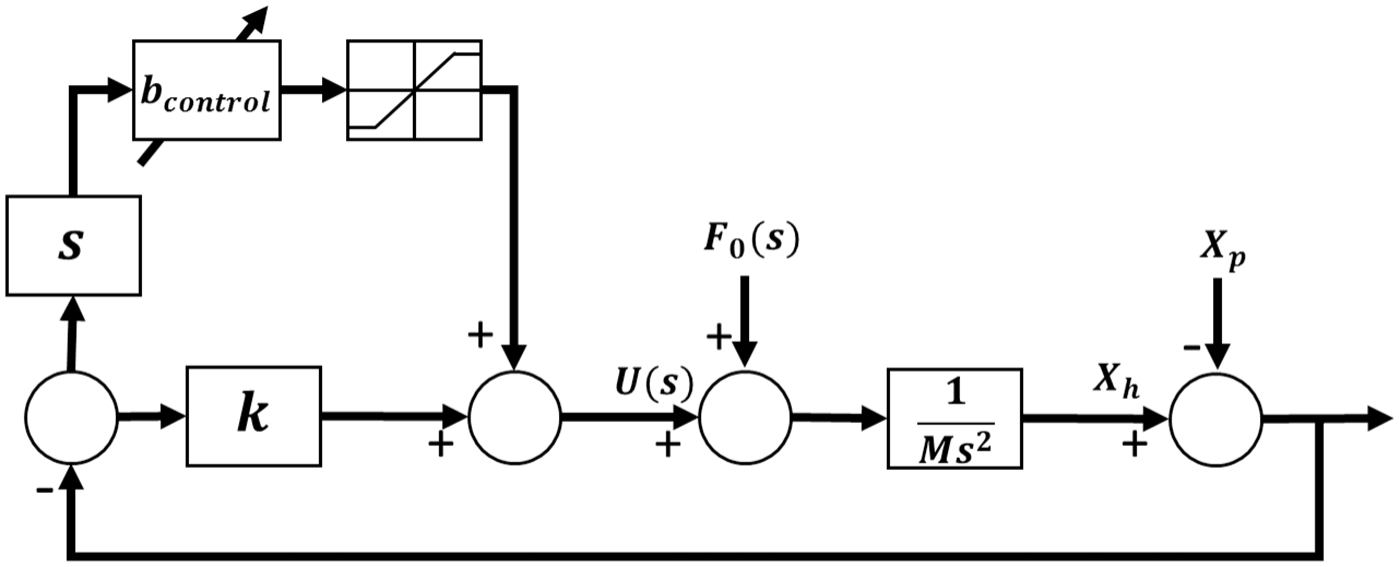
Control diagram of SAHC.

A scheme of a SAHC is shown in Fig 1, where a classical hydropneumatic suspension system is transformed into a SAHC by adding a servo-valve in between a gas accumulator and an oil cylinder. The valve orifice can be modified to obtain the desired system damping, which introduces a variable force that allows it to design a semi-active control to improve the compensator performance. A SAHC with a servo-valve is proposed in [27]. That is, when the sprung mass changes, the control system also changes automatically adapting the damping value in order to maintain the cuttoff frequency constant. The SAHC frequency response is satisfactory, but the compensator accumulator volume is much larger (99*m*^3^ and 138*m*^3^) than usual values employed in such applications about 13*m*^3^. In [17], the damping is calculated in function of displacement and drill string mass, getting an acceptable frequency response with a commercial volume accumulator of 14*m*^3^. The control diagram of SAHC is shown in the Fig 4.

The SAHC consumes less energy than the active, since external energy is required only to drive the servo-valve. This technology allows maintaining an acceptable performance and reference position when the drill string mass changes.

The next section is dedicated to the PHC when the sprung mass changes. Moreover two semi-active controllers are proposed, the first is in function of the mass and the second is in function of the mass and displacement.

The signals required by the controllers are the sprung mass value (measured on the crown block), the displacements and speeds of platform and sprung mass. These other signals can be measured via an inertial measurement unit (IMU) and the control algorithms may be implemented in a programmable logic controller (PLC).

The control requirements are to maintain the maximum gain and the cutoff frequency. Two set of design constraints are considered. Firstly a strict requirement (3dB of maximum gain and cutoff frequency of 0.056*Hz*), then a less strict requirement (10dB of maximum gain and cutoff frequency of 0.056*Hz*). The strict requirement is used in [17] and [27], to obtain a flat passband. The less strict requirement is applied in [2] and reproduced here for comparison purposes.

### Changing the sprung mass

Sprung mass variation is important to consider because offshore drilling allows a significant mass change. The drilling process starts when the drill bit touches the sea bed. In pre-salt drilling the typical depth is 2*km* and the sprung mass is approximately 150*tonnes*. The drilling process begins with this depth and finishes when the pre-salt well is reached, with 8*km* and sprung mass of about 350*tonnes*.

Gas compression, by new loading, takes place rather slow and the new pressure level is maintained over a longer period, so in this case, an isothermal change of state according to Boyle-Mariotte can be assumed [28]. The performance of a loading compensator is modified because of change in parameters such as the accumulator oil volume, the static pressure of accumulator and cylinder change. The change of pressure is shown in (see Eq (9)) and the volume change is:

$$\begin{array}{c} \begin{array}{c} V_{G0}=V_{0}\frac{M_{0}}{M} \end{array} \end{array}$$ (23)

Using the Eqs (14), (18) and (9), the expression of natural frequency is obtained in function of the sprung mass(*M*).

$$\begin{array}{cc} & \omega_{n}=\sqrt{\frac{Mg+P_{atm}A}{V_{G0}M}}\\ & \zeta =\frac{b+c}{2\omega_{n}M} \end{array}$$ (24)

This frequency is proportional to square root of the sprung mass. The damping is inversely proportional to the mass. This means that, the dimensionless factor *l* is proportional to the mass (look at Fig 2B). The cutoff frequency depends on the natural frequency and the factor *l*, for this reason, the relation with the sprung mass is not evident.

### Variable damping in function of the mass

In order to enhance the vibration control, the overall damping of a system may be designed according to other system parameters, such as masses (spring and unsprung), stiffness and damping of the corresponding structure. Based on a decentralized static output-feedback *H*_∞_ controller, [29] presents as design of additional dampers for a passive energy dissipation system for large structures.

For the offshore drilling operation, the sprung mass varies according to the length of the drill string, therefore a semi-active solution is preferred in this work.

The semi-active control uses just one servo-valve, which only acts when the sprung mass changes. This solution is simple and robust, ensuring the safety operation even in the context of mechanical/electrical failures, because the valve position is kept at the last controlled level (proportional to the mass), thus holding the system damping at a level close to the desired one.

The servo-valve generates a damping *b* for each value of sprung mass to maintain a constant damping ratio *ζ*, so the frequency response of transfer function of Eq (17) respects the cutoff frequency and the maximum gain. Using the Eq (18), the equation to maintain constant damping *ζ* is obtained, as follows:

$$\begin{array}{c} b_{pas}\left(M\right)=2\zeta \omega_{n}M-c \end{array}$$ (25)

### Damping variable in function of the system displacement and the mass

The semi-active control uses just one servo-valve, as the previous control, but this valve modifies the system damping in high frequency to optimize the compensator performance and to reduce the accumulator volume. A parallel redundant system in case of a servo-valve failure can also allow the drilling process to continue in a simple and robust manner (normally, closed servo-valve).

The servo-valve modifies the damping *ζ* in function of displacement and sprung mass. In [17] the following control law is used:

$$\begin{array}{c} b_{sky}\left({\dot{x}}_{p},M\right)=b_{1}\left(M\right)+b_{2}\left(M\right)\frac{{\dot{x}}_{p}\left(t\right)}{{\dot{x}}_{p}\left(t\right)-{\dot{x}}_{h}\left(t\right)} \end{array}$$ (26)

This control is a kind of continuous skyhook [30], but the closed loop transfer function has one zero. The parameters *b*_1_ and *b*_2_ are 15% and 85% of the desired value by classical skyhook, the zero is obtained 6 times larger than the real part of desired poles.

$$\begin{array}{c} \begin{array}{c} b_{1}\left(M\right)=2\zeta \omega_{n}M\left(0.15\right)-c\\ b_{2}\left(M\right)=2\zeta \omega_{n}M\left(0.85\right) \end{array} \end{array}$$ (27)

**Fig 5:**
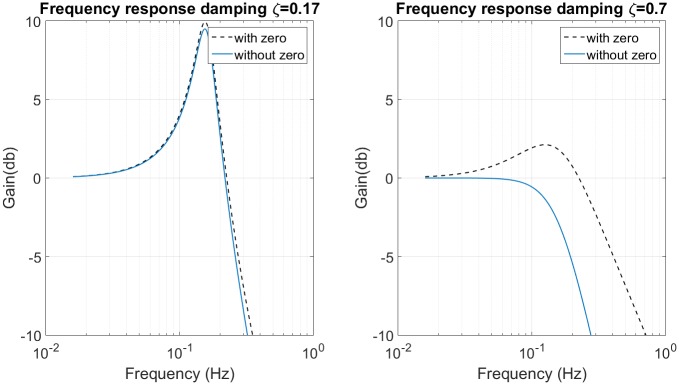
Skyhook effect. (a) Low damping value. (b) High damping value.

The advantage of skyhook is to cancel the zero performance in transmittance transfer function of PHC Eq (28). This works successfully when the desired damping has high value and the performance is improved (look at Fig 5B). However, when the desired damping is small, the compensator performance with and without zero are similar. Fig 5 illustrates the frequency response of the compensator with and without zeros for two different damping ratios, *i.e.* *ζ* = 0.17 in Fig 5A and *ζ* = 0.7 in Fig 5B.

$$\begin{array}{c} \frac{x_{p}\left(s\right)}{x_{h}\left(s\right)}=\frac{\left(\frac{b_{1}\left(M\right)+c}{M}s+\frac{k}{M}\right)}{\left(s^{2}+\frac{\left(b\left(M\right)+b_{1}\left(M\right)+c\right)}{M}s+\frac{k}{M}\right)} \end{array}$$ (28)

In [17] the skyhook response has a flat passband and a cutoff frequency of 0.056*Hz*, however, it presents low attenuation in transition band. When the platform is moved by the ocean wave, the attenuation rate is 75%.

The skyhook reaches the desired frequency response with a gain of 10*dB* and a damping (*ζ*) of 0.17. However, for small damping coefficient the performance between the function with and without zero is similar and the required volume with skyhook is similar to the required accumulator volume for variable damping in function of mass. Furthermore, the skyhook requires measurements of absolute velocity as well as relative velocity. Accurate measurement of the absolute velocity may be difficult to achieve.

The balance control is a strategy easier to implement because it uses a directly measurable relative position and relative velocity signals, as shown in Eq 29.

$$\begin{array}{c} b_{bal}\left({\dot{x}}_{p},M\right)=b_{d}\left(M\right)+\left(k_{d}\left(M\right)-k\left(M\right)\right)\frac{x_{p}\left(t\right)-x_{h}\left(t\right)}{{\dot{x}}_{p}\left(t\right)-{\dot{x}}_{h}\left(t\right)} \end{array}$$ (29)

The desired parameters *b*_*d*_ and *k*_*d*_ are calculated in function of the sprung mass and the desired natural frequency *w*_*n*_, which is calculated with the desired value of cutoff frequency Eq (16). The stiffness value *k*_*d*_ is designed to be 10% of the stiffness obtained with the desired natural frequency. This gives the best results based on conditions of actuator saturation. The desired damping *b*_*d*_ is obtained in the same way as the variable damping in function of the mass Eq (25).

$$\begin{array}{ccc} k_{d}\left(M\right) & = & 0.1{\left(\omega_{n}\right)}^{2}M\\ b_{d}\left(M\right) & = & 2\zeta \sqrt{k_{d}M}-c \end{array}$$ (30)

A similar control is the traditional continuous balance, proposed by [28], its expression is:

$$\begin{array}{c} b_{Tr-Bal}\left({\dot{x}}_{p},M\right)=-k\left(M\right)\frac{x_{p}\left(t\right)-x_{h}\left(t\right)}{{\dot{x}}_{p}\left(t\right)-{\dot{x}}_{h}\left(t\right)} \end{array}$$ (31)

and its goal is to reduce the acceleration, this is achieved as follows, the damper and sprung forces should have the same order of magnitude. So the acceleration of the sprung mass is reduced. However, the objective in this control is reduced the desired frequency response of displacement.

The stability semi-active control systems primarily acts to oppose the motion of the structural system promoting the global stability of the structure (for more information see [8]). The S3 Appendix) presents the stability analysis for the proposed semi-active controllers skyhook and balance. The proof considers the system dynamics, the bounded perturbation and the semi-active control command saturation. This proof uses the Lyapunov stability theory, which determines that the system is globally uniformly ultimate bounded.

## Numerical simulations

Four subsections describe the obtained results. Firstly, a PHC is designed with the proposed methodology to obtain the desired frequency response. Second, the bulk modulus influence is determined with the condition to neglect the bulk modulus. Third, the damping control in function of the mass 10*dB* is applied in the SAHC that was designed with the methodology (accumulator volume of 99*m*^3^) and the control of 3*dB* in another compensator with an accumulator volume of 138 *m*^3^. Finally, the balance and skyhook controls are used in SAHCs with accumulator volumes of 49.5*m*^3^ and 18.5*m*^3^, respectively.

### Dimensionless factor results

Using the methodology presented in subsection PHC design (see Fig 3), it is possible to define the physical parameters of PHC. The frequency and physical parameters are defined and they are used to calculate the remaining physical parameters.

The conditions for PHC design are: cutoff frequency of 0.056*Hz*, cutoff frequency gain of -3*dB* and maximum gain of 10*dB*. Using Fig 2A we obtain the damping coefficient value of 0.17. Thus, using the Fig 2B we obtain the dimensionless factor.

The compensator is designed with the maximum mass (350*tonnes*), the cylinder viscous friction is 1000*Ns*/*m*, the atmosphere pressure is 0.1*MPa* and the maximum pressure is 22.8*MPa*. This value is close to values found on literature, 26.6*MPa* in [2] and 21.0*MPa* in [31]. The cylinder area is calculated with Eq (22), its value is approximately 0.15*m*^2^.

The final step is to use the physical and the frequency parameters to calculate with Eq (18) the accumulator volume 42.8*m*^3^, accumulator stiffness 17.2*kN*/*m* and the valve damping 25.7*kNs*/*m*. Subsection *Mass* *varying* *semi*—*active* *results* shows the performance of the designed PHC, with the desired cutoff frequency and the maximum gain of 10*dB*.

### Bulk modulus effect

The PHC was designed without considering the bulk modulus. Now, its influence on frequency response is addressed. The simulation uses an oil volume of 0.153*m*^3^ and a bulk modulus of 0.3*GPa*, this is the lowest value found in [19] and represents the bulk modulus with 2% entrained air, which is a small value because the typical value without entrained air is 1.7GPa. The entrained air increases the bulk modulus effect in the frequency response.

**Fig 6:**
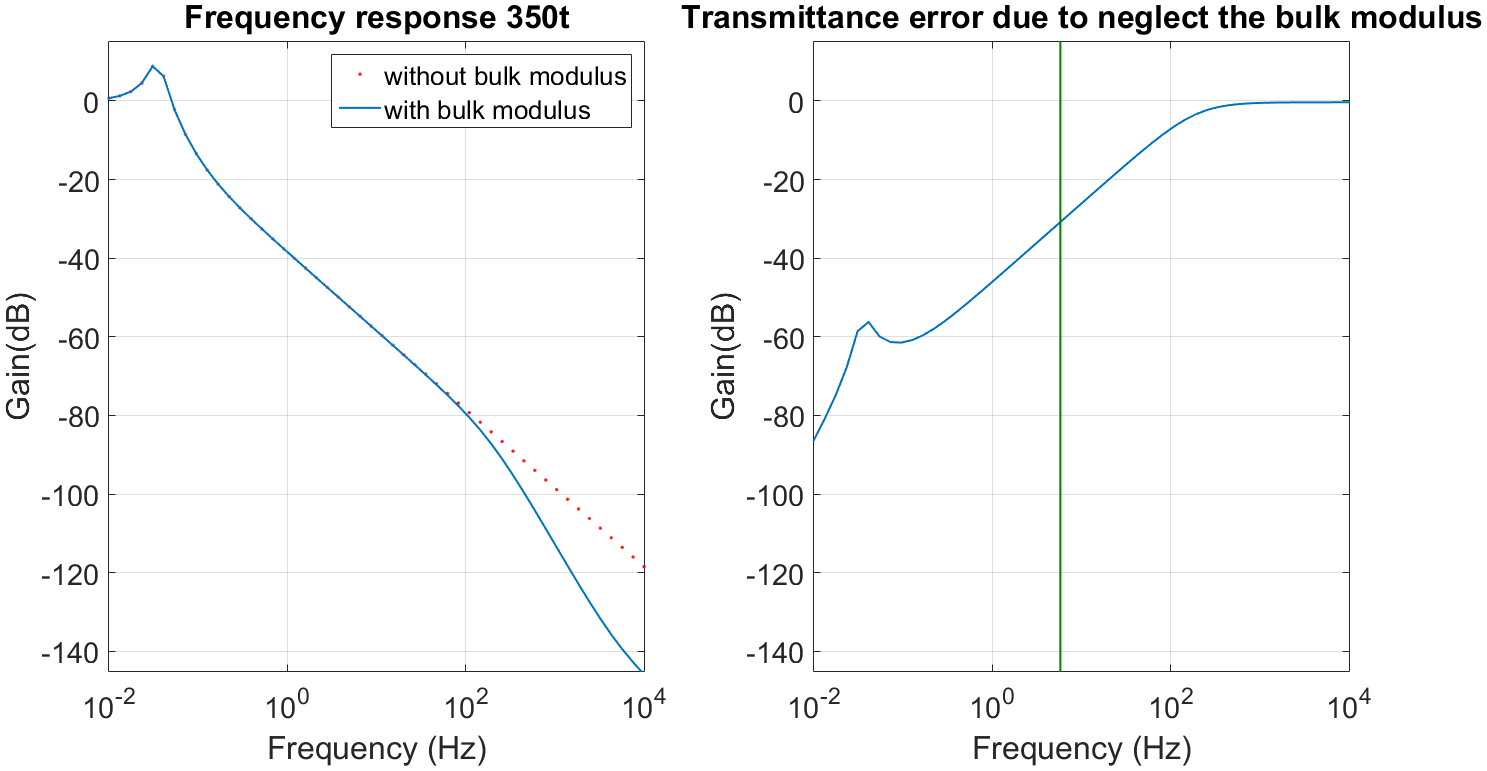
Bulk modulus results. (a) Frequency response with and without bulk modulus for 350t. (b) Normalized transmittance error without bulk modulus.

The previous condition to choose the model with and without the bulk modulus is tested. Firstly, the frequency *s*_*b*_ is calculated with Eq (15), that represents the maximum frequency where the impedance simplification is valid and the bulk modulus can be neglected, this value is 6*Hz*. The vertical line of Fig 6B represents *s*_*b*_, the transmittance error is approximately 3% (30 *dB*). The relative error transmittance is obtained with the Eqs (4) and (13). The frequency range of interest is between 0.056*Hz* and 0.3*Hz*, most energy of Brazilian ocean waves is distributed in this range, and the simplification is valid below to the value of 6*Hz*, therefore the bulk modulus is neglected for the PHC.

Fig 6A shows the frequency response of PHC with and without bulk modulus, these are obtained with Eqs (4) and (13), respectively. The difference between the responses below 10*Hz* can be neglected.

### Mass varying semi-active results

The semi-active control in function of the mass is applied in the compensator. Its performance is evaluated with the frequency criteria.

#### Cutoff frequency

*ω*_*c*_ ≤ 0.056*Hz*, since the energy of ocean waves are concentrated in frequencies larger than this value.

#### Maximum gain of frequency response

The ideal response has a maximum gain of zero, which means that the compensator never amplifies the input movement. Maximum gain greater than 0*dB* is acceptable for low frequency (*ω* ≤ 0.056*Hz*), since the waves have little energy in this range. The smaller the maximum gain, the better in the performance.

#### Attenuation rate relative to sea condition 4

A motion platform caused by an ocean wave of sea condition 4 is taken from [2], The significant wave height and the frequency spectrum of this wave is distributed around 0.14Hz, which is acceptable in Brazilian case. This attenuation is the most important criteria, because it represents the attenuation rate for an ocean wave, which is characterized by many waves with different frequencies and amplitudes.

#### Frequency response gain at 0.17*Hz* of frequency response

This frequency value is important, because the maximum energy of ocean wave is distributed nearby this value, for sea condition 4, the wave energy reaches to maximum around 0.13*Hz* [2]. Therefore, the gain in this frequency means the attenuation of the ocean waves of maximum energy. The higher attenuation means a better control performance.

#### Maximum accumulator volume

The PHC is designed for each semi-active control case to have the desired performance. Thus, four compensators are designed with the same cylinder area, but with different accumulator sizes. The accumulator size is important to determine if the compensator is feasible.

The control with the desired frequency response of 10*dB* maximum gain and cutoff frequency of 0.056*Hz* is compared with the control of 3*dB* maximum gain and the same cutoff frequency (more strict conditions). The compensator is adapted by the servo-valve controller for the mass interval of 150*tonnes*-350*tonnes*. The damping *b*_*pas*_ is calculated with Eq (25).

**Fig 7:**
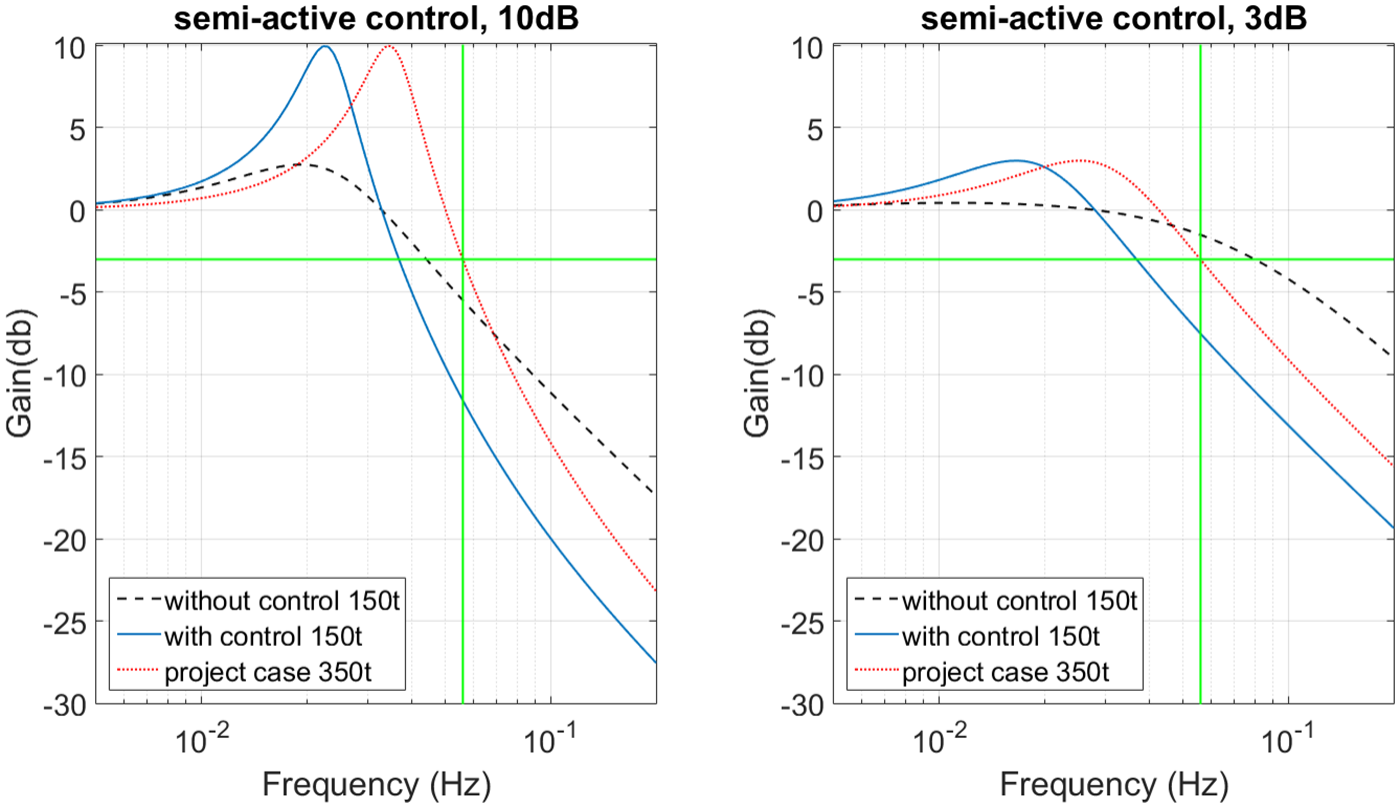
Frequency response semi-active control in function of mass. (a) Control with maximum gain of 10*dB* (b) Control with maximum gain of 3*dB*.

Fig 7A shows the responses for the control with 10*dB* for:

- The maximum mass without control
- The minimum mass with control and without control

The maximum mass does not need a control, because the PHC is designed to work with this mass. The compensator has a maximum volume of 99*m*^3^ when it supports a minimum mass. The desired frequency response requirement is reached with the control. The transmittance gain for a sinewave with period of 5.8*s* (0.17*Hz*) is -25.9*dB* with control and -16*dB* without control, so the control improves the attenuation from 85% to 95% in this frequency. A better performance with control in the transition band is explained by the damping ratio (*ζ*) value, which is 0.41 without control in 0.17 with control.

Fig 7B presents the control responses for 3*dB* maximum gain with a damping *ζ* of 0.54. Compared with the 10*dB* control, the 3*dB* control has a better passband, but a lower attenuation in the transition band. Additionally, 3*dB* control has an attenuation for sinewave with period of 5.8*s* between 81% and 88%, while the smaller attenuation for 10*dB* maximum gain control is 86%, for the minimum sprung mass. Moreover, the maximum volume is 138*m*^3^ for 3*dB* control, the volume is 29% reduced in the 10*dB* control. The main parameters of Fig 7 are summarized in Table 1.

**Table 1:** Summary of frequency response for SAHC in function of sprung mass.

Maximum designed gain	10 dB			3dB		
Semi-active control	without		with	without		with
M(*tonnes*)	350	150	150	350	150	150
V (*m*^3^)	42.8	99.9	99.9	59	138	138
*ω*_*c*_ (*Hz*)	0.056	0.045	0.037	0.056	0.08	0.037
Maximum gain (*dB*)	10	2.5	10	3	0.4	3
Gain for 0.17*Hz*(*dB*)	-21.3	-16.0	-25.9	-14.2	-7.7	-17.8

**Fig 8:**
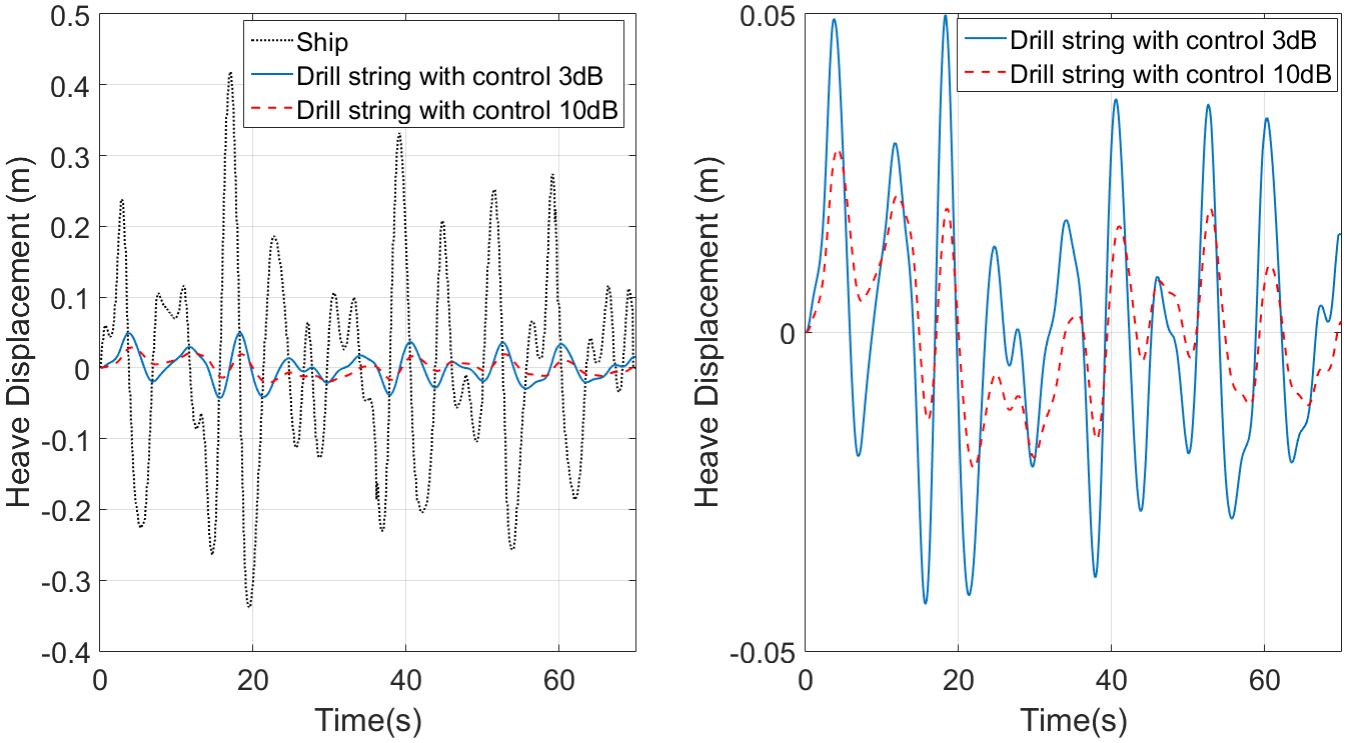
Compensator performance for random ocean wave. (a) Input signal and semi-active control responses for 150*tonnes* mass. (b) Responses of 3*dB* and 10*dB* semi-active controls.

The control responses for sprung mass of 150*tonnes* when the platform is moved by the ocean wave is shown in Fig 8A, this motion platform is taken from [2]. The significant wave height and the frequency spectrum of the wave energy are relative to sea condition 4 and the wave has a spectrum distributed around 0.14*Hz*, which is acceptable in Brazilian case. The Fig 8B also shows the control response with 3*dB* and 10*dB* maximum gains for the platform movement. The Fig 8B focus exclusively on the responses. The control with 3*dB* reaches an attenuation of 88% and with 10*dB* the attenuation is 93%, for a sprung mass of 150*tonnes*. When the sprung mass is 350*tonnes*, the attenuation rates are 83% and 88%, respectively. In [2], an attenuation of 83% is described as an excellent performance.

### System displacement and mass varying semi-active results

The compensator is redesigned with a maximum accumulator volume of 49.5*m*^3^ (cylinder area of 0.16*m*^2^) which is half of the volume required with the semi-active control in function of the mass. The balance control uses Eq (29) with *ζ* of 0.25 (maximum gain of 7*dB*). The valve has a diameter of 0.016m and 0.069m in opened and closed states, respectively. Then, the damping coefficient value is between 2*MNs*/*m* and 0*MNs*/*m*, these are the saturation values.

The main remark on feasibility is obtained by comparing the semi-active actuator requirements of SAHC with the semi-active actuators used by structural control. The heave motion in ocean waves has a slow dynamic with frequencies between 0.06*Hz* and 0.21*Hz* and the wave considered in structural control are between 0.4*Hz* and 5.3*Hz*. The force magnitudes developed by the semi-active actuators of structures are similar to the forces required by the heave compensator, the semi-active structural actuators developed a force magnitude between 2*kN* and 1*MN* [8] and the magnitude force assumed in this article is 2*MN*.

**Fig 9:**
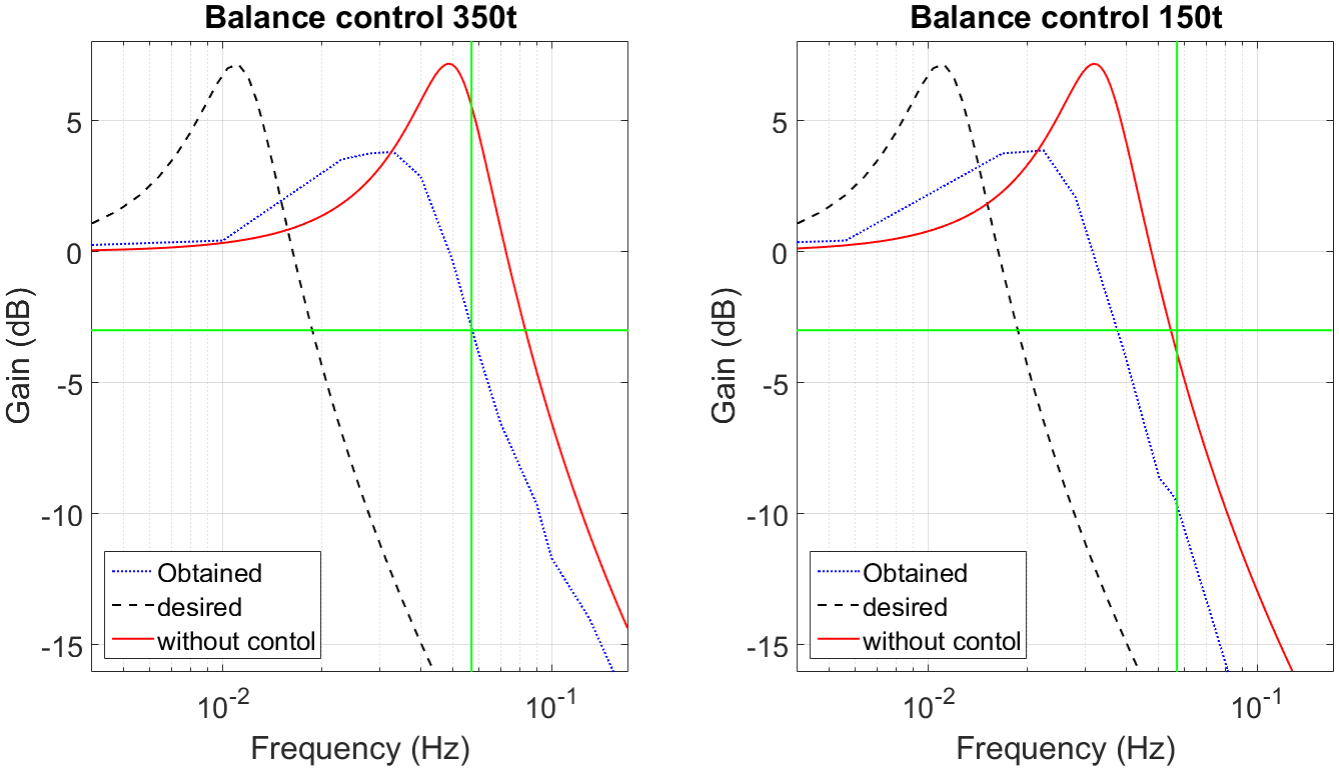
Balance control response. (a) Sprung mass of 350*tonnes*. (b) Sprung mass of 150*tonnes*.

For each sprung mass (150*tonnes* and 350*tonnes*), Fig 9 shows: the desired performance of balance control, the SAHC performance with the balance control (considering servo-valve saturation) and the performance without control. The latter uses a constant damping, which is calculated to obtain the same maximum gain of desired response. These responses are obtained for the minimum and the maximum sprung masses.

The model of SAHC is simulated in Simulink with a sinusoidal input of amplitude 1*m* and a frequency value between 0.005*Hz* and 1.1*Hz*. The frequency value is constant during each simulation. The simulation is repeated with a different input frequency, this frequency and the output amplitude are registered to plot the obtained frequency response of balance and skyhook controls.

The desired frequency response is different from the frequency response obtained with balance control, the maximum gain is 3.9*dB* and the desired 7*dB*, the natural frequencies and damping are larger than the desired values. However, the cutoff frequency (0.056*Hz*) is respected and the attenuation in 0.17*Hz* is between 84% and 83%. This is a small value, because the desired attenuation in this frequency is 97%. The compensator with 150*tonnes* could be used without the balance control, but an increase in sprung mass increases the compensator‘s cutoff frequency than 0.056Hz. This amplifies ocean waves. The frequency response data is summarized in Table 2.

**Table 2:** Summary of frequency response for balance control.

Semi-active control	without		Desired	Balance obtained	
Mass (*tonnes*)	150	350	150	150	350
V (*m*^3^)	49	21	49	49	21
*ω*_*c*_ (*rad*/*s*)	0.055	0.091	0.018	0.039	0.056
Maximum gain (*dB*)	3	3	7	3.9	3.9
Gain for 0.17*Hz* (*dB*)	14	-19	-29	-23	-16

**Fig 10:**
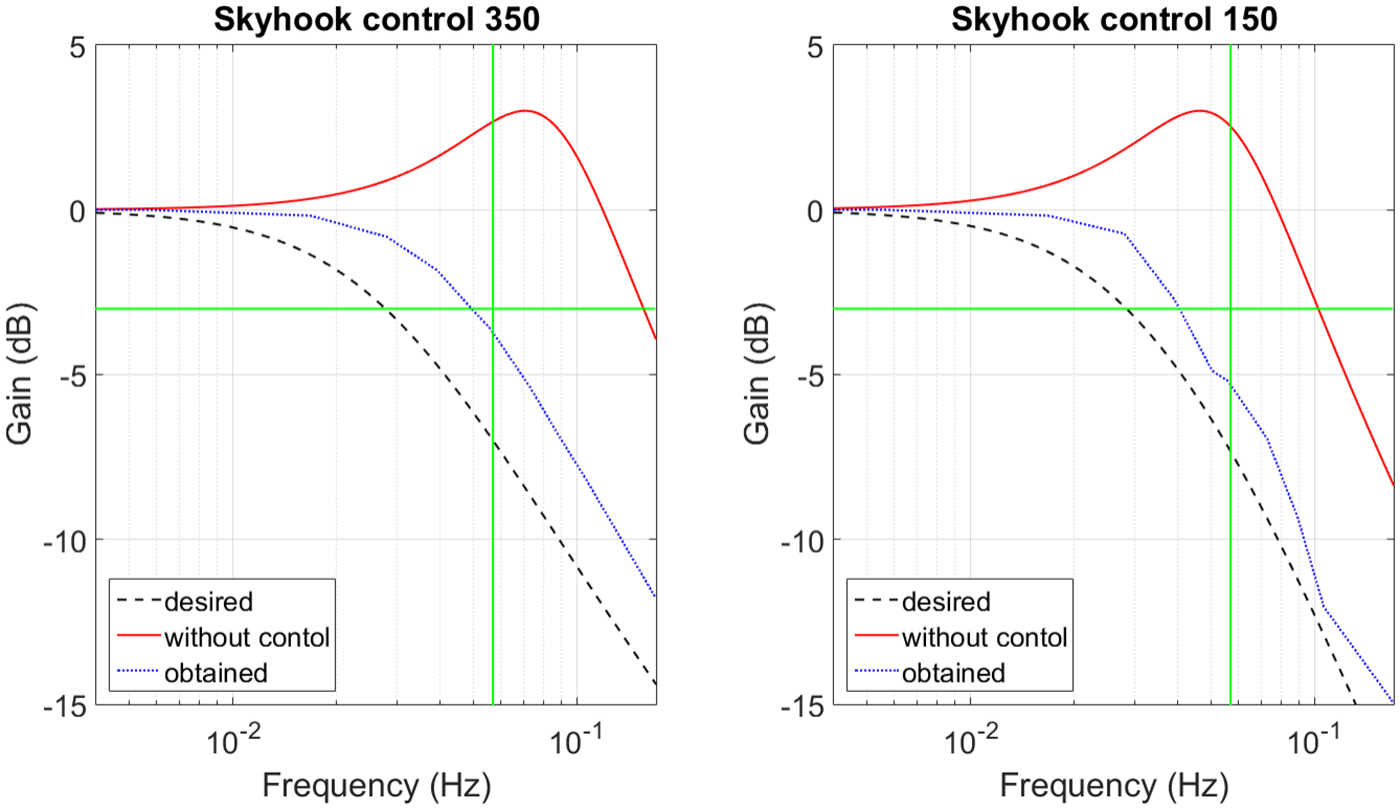
Skyhook control response. (a) Sprung mass of 350*tonnes*. (b) Sprung mass of 150*tonnes*.

A skyhook control of Eq (26) is used in [17]. The compensator was designed with the maximum accumulator volume of 18.4*m*^3^, cylinder area of 0.16*m*^2^ and saturation between 2*MNs*/*m* and 0*MNs*/*m* (the same used in balance control). For the maximum and minimum sprung masses, Fig 10 shows: the desired response of skyhook control, the actual response obtained for skyhook control (considering the saturation) and the response without control.

The frequency response obtained has the desired flat passband and the desired cutoff frequency, however the attenuation in the transition band is different. The attenuation obtained is between 74% and 80% for a frequency of 0.17*Hz* and the desired attenuation is between 75% and 83%. The response without control amplifies the sprung mass movement and has a cutoff frequency of 0.09*Hz* (for a sprung mass of 150 *tonnes*) and 0.15*Hz* (for a sprung mass of 350*tonnes*), so the control advantage is to ensure that this movement is always reduced, but the reduction is smaller than 80%. This small reduction in the transition band is due to the servo-valve saturation and the high damping used in skyhook. Fig 5 shows that the skyhook should have a high damping to obtain better results than the passive system. Table 3 summarizes the frequency response data of Skyhook control.

**Table 3:** Summary of frequency response for skyhook control.

Semi-active control	without		Skyhook desired		Skyhook obtained	
Mass(*tonnes*)	150	350	150	350	150	350
V (*m*^3^)	18.4	7.9	18.4	7.9	18.4	7.9
*ω*_*c*_(*Hz*)	0.098	0.151	0.028	0.02	0.039	0.050
Maximum gain (*dB*)	3	3	0	0	0	0
Gain for 0.17*Hz* (*dB*)	-8.7	-4.0	-17.5	-13.4	-14.0	-11.7

**Fig 11:**
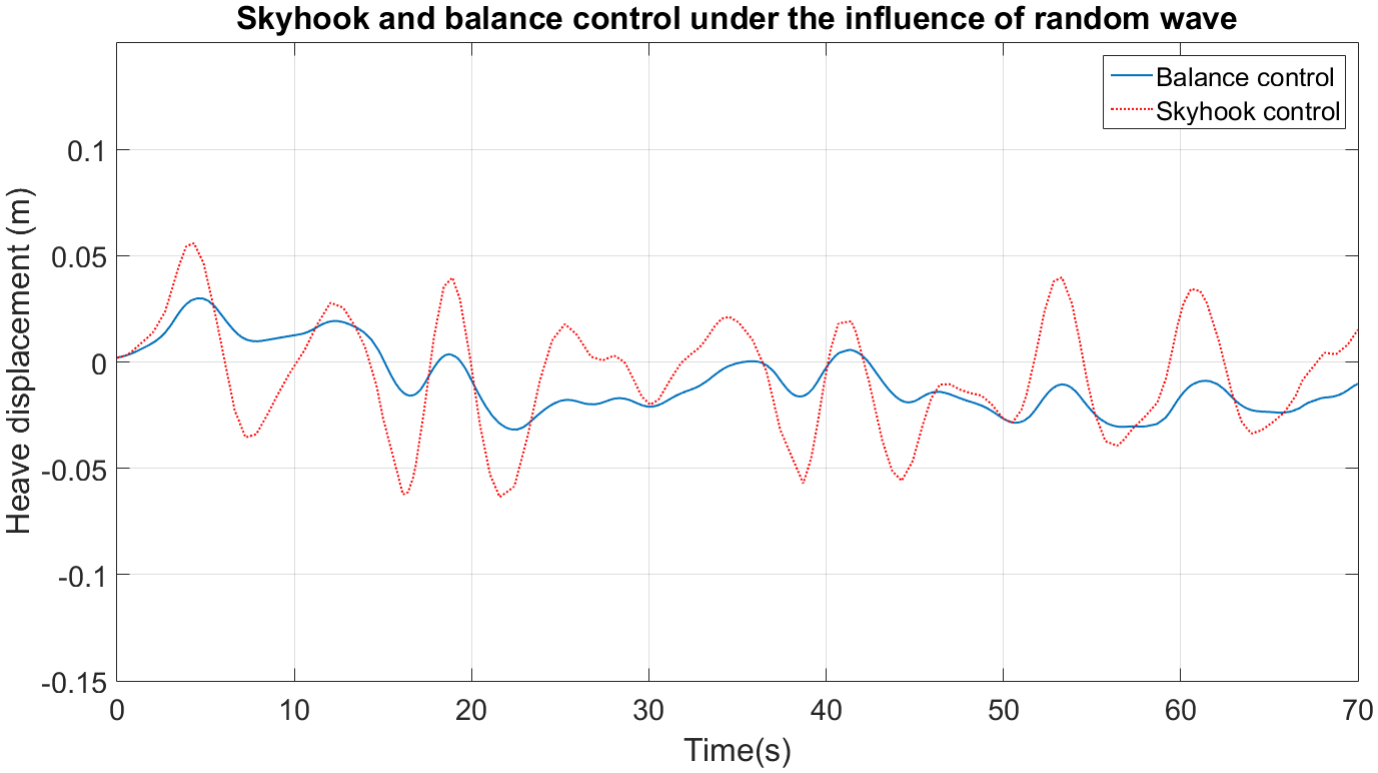
Compensator performance for random ocean wave with skyhook control and balance control.

Table 4 compares the performance and the physical requirements of the four SAHC designed here and a commercial AHC [31]. This AHC has an attenuation larger than 95% for any ocean wave, also its accumulator volume is between 7*m*^3^ and 13.5*m*^3^, depending on sprung mass. The control of 10*dB* has an acceptable attenuation rate (93%), but the accumulator volume is huge (99*m*^3^) and it must be used for cases of ocean wave with frequency larger than 0.056*Hz*. The compensator of 3*dB* has the largest volume (138*m*^3^), its attenuation is 83% and it never amplifies the input displacement. The skyhook and balance control have similar attenuation (87% 90%), this is shown in Fig 11, which uses the ocean wave of Fig 8A as input. In theory, the balance control has a better performance and larger volume than the skyhook presented in [17]. But, due to the saturation of actuator both systems have similar performances and the skyhook control needs an accumulator volume smaller than the volume used by the balance control.

**Table 4:** Control systems comparison for sprung mass of 150*tonnes*.

Control	skyhook	Balance	10dB	3dB	AHC
Maximum volume (*m*^3^)	18.4	49	99	138	13
Attenuation rate relative to sea condition 4(%)	87	90	95	83	95
Maximum gain (*dB*)	0	7	10	3	-
Minimum attenuation at 0.17*Hz*(%)	80	93	86	81	95

The robustness of the controllers in terms of changes in the parameter is discussed in S2 Appendix, the suspended mass and damping of the servo-valve are the changing parameters, with 10% variation of real value. The controllers are not fully robust, because there are changes in frequency responses. The maximum gain with parameter variation is 25% for balance control, 13% for semi-active control of 10*dB*, 8% for semi-active control of 3*dB* and the skyhook control has no variation. However, the cutoff frequency is respected and the attenuation variation of 0.17*Hz* is less than 8%, except the balance control, its variation is 20%.

Skyhook control presents a better response in terms of parameters change. Its frequency response with the parameter variation is almost the same as the response without variation. The frequency response of all the presented controllers are still acceptable and better than without control (see S2 Appendix).

Mechanical SAHC designed in [10] has an attenuation similar to AHC, which is larger than 95% with an accumulator volume of 10*m*^3^ and energy consumption in 60s of 2.8*MJ* that is 11.2% of the AHC energy consumption. [10] also shows the SAHC energy consumption of 3,5*MJ* that is 14.2% of the AHC energy consumption. The experiment presented in [12] shows the SAHC energy consumption approximately 33% of AHC energy consumption and its attenuation is better than 95%. Theses SAHC has a similar attenuation of AHC and the energy consumption is considerably reduced. The SAHC presented here has insignificant energy consumption, however its attenuation should be increased.

## Experimental validation

Laboratory experiments and simulators have been essential for the development of offshore drilling, providing answers to industral and academic questions on drilling vibration mitigation and associated issues. It is important to note that full-scale field trials could be impractical or impossible [32].

In this section, the behaviour of the prototype, concerning the variation of mass and damping, is evaluated experimentally. The objective is to show the frequency response of the prototype for different configurations of mass and valve position (damping).

### Prototype

**Fig 12:**
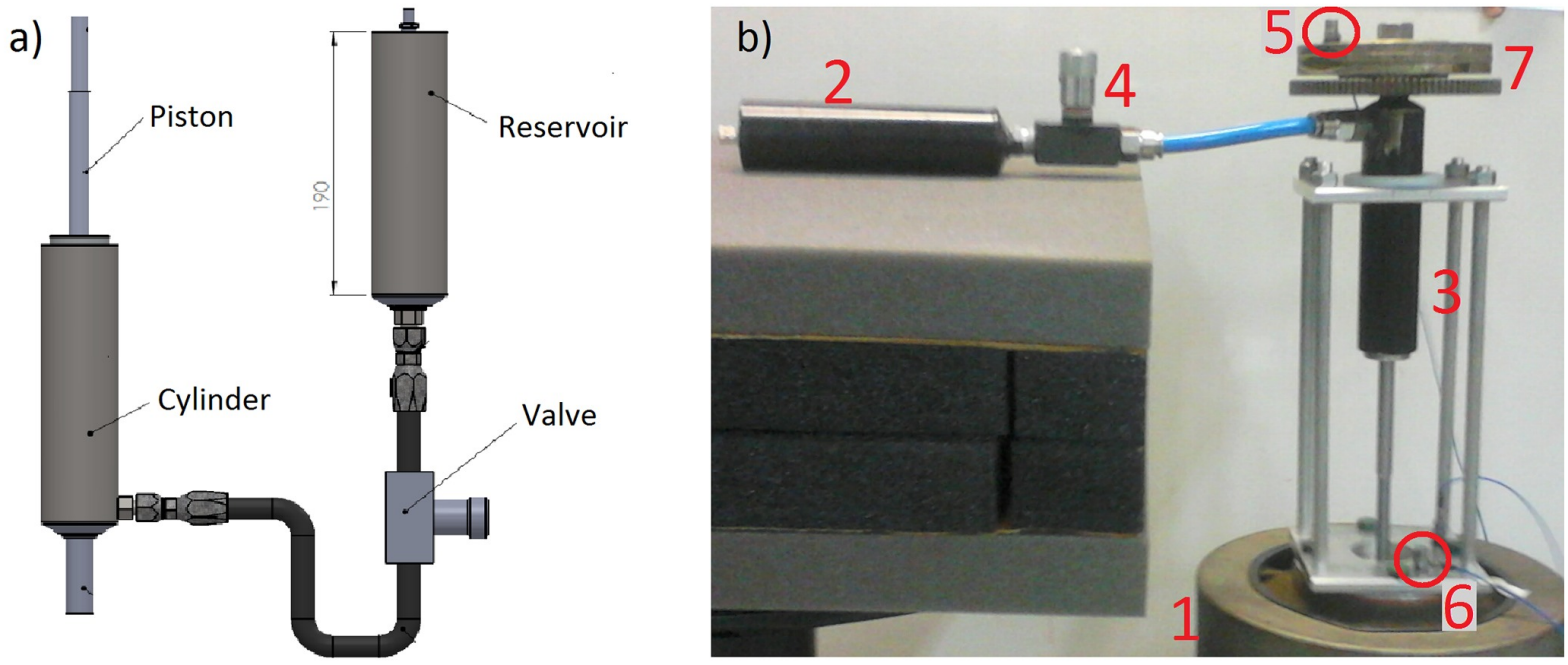
Setup. (a)Schematic illustration of the prototype. (b) Experimental setup.

Fig 12 presents a schematic illustration of the prototype.

Table 5 presents the components of the experimental setup from Fig 12B.

**Table 5:** Experiment setup.

Number	Description
1	Shaker Labworks model ET-127.
2	Gas reservoir.
3	Cylinder.
4	Manual Parker *Colorflow*^®^ Control Valve which is used three valve positions.
5	Upper accelerometer PCB PIEZOTRONICS 352C33(sensitivity 100.5mV/g) that measures the output signal.
6	Lower accelerometer PCB PIEZOTRONICS 352C03(sensitivity 10.27mV/g) that measures the input signal.
7	Sprung mass.

**Fig 13:**
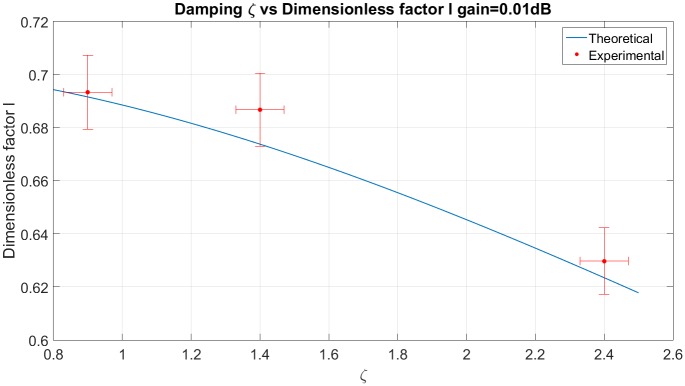
Dimensionless factor vs. *ζ* and its confidence interval for experimental setup.

The parameters of the compensator are calculated using the methodology proposed in the section *PHC* *design*. The required frequency response has cutoff frequency 27.5*rad*/*s* with −0.1*dB* of gain and *ζ* of 0.9. Fig 13 provides the theoretical dimensionless factor *l* used to design the compensator and the corresponding experimental *ζ*. The confidence interval was computed considering a precision estimation of 5% for *ζ* and 2% for *l*.

Pressure is limited to 150*kPa* and the maximum mass is 8.5*kg*. Based on these information, Eq (22) is used to calculate a cross sectional area of 0.0017*m*^2^ for the prototype. Moreover, *b*, *k* and *V*_*G*0_ are calculated using Eq 18.

The first column of Table 6 shows the parameters of the prototype obtained using the methodology. Changing the sprung mass to 6*kg*, Table 6 presents the parameters of the prototype for this new mass. These parameters are calculated using the Eqs (23) and (24).

**Table 6:** Theoretical parameters for pipe length of 200mm.

Mass	8kg	6kg
*ζ*	1.4	1.7405
*b*(*Ns*/*m*)	430.67	430.67
*ω*_*n*_(*rad*/*s*)	18.0956	19.0337
*K*(*N*/*m*)	2783.3	2354.8
*V*(*l*)	0.2045	0.2223

The dimensions of the compensator are determined by using the parameters calculated. The cylinder has the length of 200*mm* and the pipe connecting the cylinder and the valve has the length of 200*mm* and the inner diameter of 12.7*mm* (1/2*inch*).

### Experimental results

The shaker cited in Table 5 is used to provide a sweep from the frequency 25 *rad*/*s* to 30 *rad*/*s*. Accelerometers are used during the sweep to obtain the displacement provided by the shaker and the displacement of the compensator response. The experimental Bode diagram is drawn form these data.

**Fig 14:**
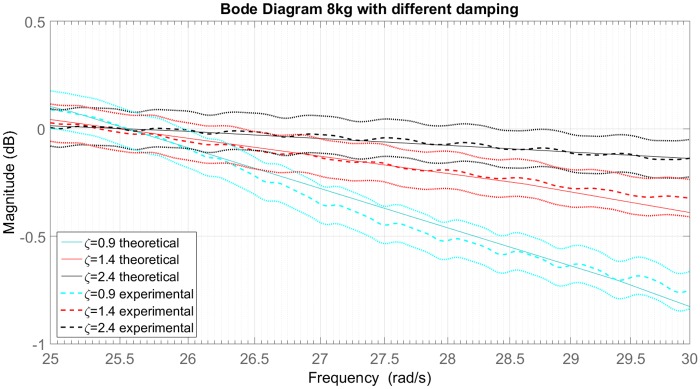
Bode diagram for 8kg sprung mass considering different dampings and their confidence intervals.

Fig 14 shows the experimental and the theoretical Bode diagram for the sprung mass of 8*kg* in 3 different closed valve positions, the confidence intervals are due to the fluctuation (about 1%) of the signal generated by the shaker. Table 7 shows the damping ratio(*ζ*) and the damping coefficient(*b*) for each valve position. Changes in the parameters *ζ* and *b* are noticed, while the accumulator volume and the cutoff frequency are constant. It is important to note that the methodology proposed is able to provide an experimental frequency response close to the expected theoretical response.

**Table 7:** Damping coefficients for sprung mass of 8*kg* and 6*kg*.

Valve Position	*ζ*	*b*(*Ns*/*m*)
1^*st*^	0.90	278.78
2^*nd*^	1.40	495.61
3^*rd*^	2.40	960.26

The dimensionless factor is obtained experimentally, using the experiment cutoff frequency and the theoretical natural frequency. Fig 13 shows the dimensionless factor for the three damping rates of Table 7 and the dimensionless factor calculated with Eq (21). The experimental results presents a correspondence with the theoretical results.

**Fig 15:**
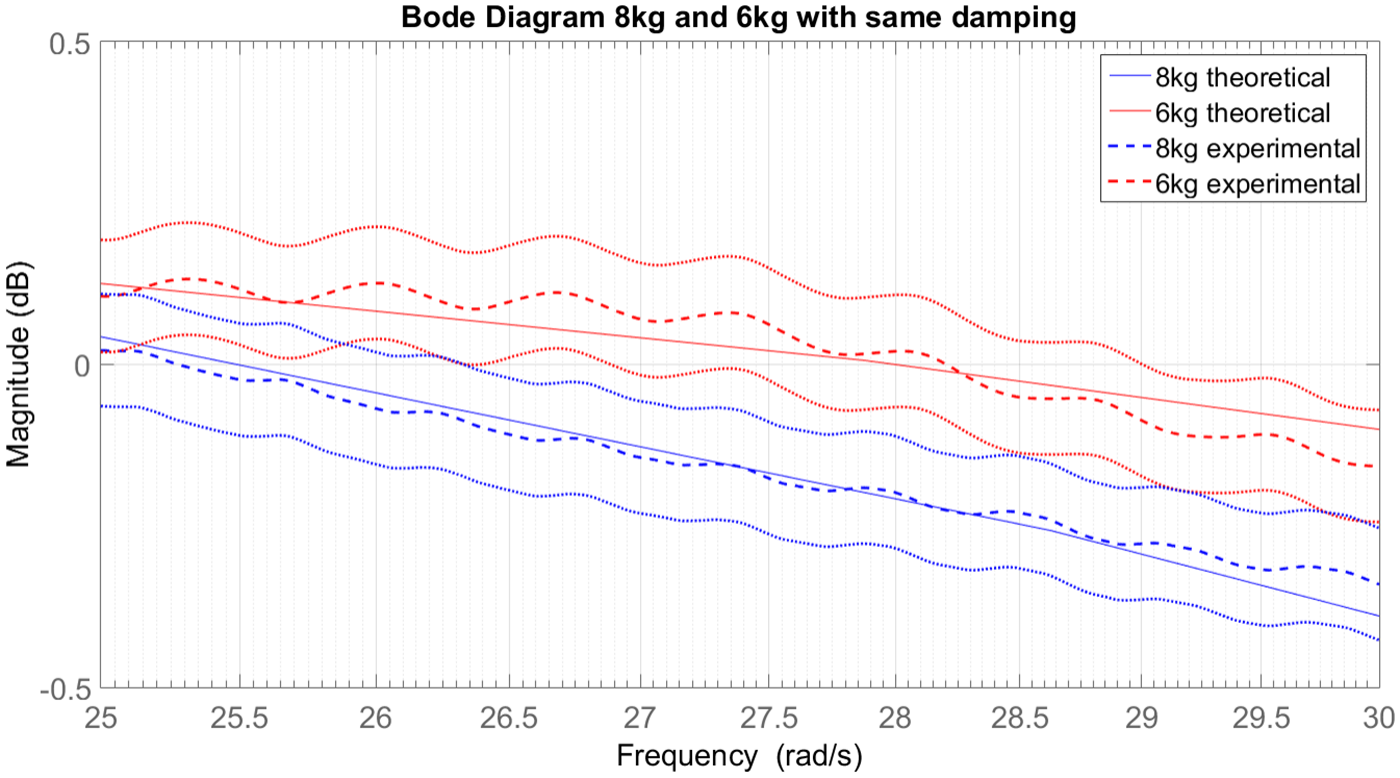
Bode diagram of 8kg and 6kg sprung masses with same damping and their confidence intervals.

Comparing Figs 15 and 7, an opposite behaviour regarding the mass change and the change in the natural frequency was found. This difference occurs due to the high difference in the cylinders’ pressures (in Fig 15 the operating pressure is close to the *P*_*atm*_).

## Conclusion

This article presented a design methodology of a hydropneumatic PHC with the desired frequency response and an associated dimensionless factor. The example designed with cutoff frequency of 0.056*Hz* and maximum gain of 10*dB* shows a direct way to implement the methodology. Furthermore, the methodology is used to design a prototype, which presents an acceptable experimental behavior.

The influence of bulk modulus on frequency response of PHC is addressed and a simple condition is found to decide whether the bulk modulus should be considered in the model. In the given example, this condition ensures a complexity reduction of the system model, thus an efficient model based control for the studied cases.

Semi-active control in function of mass is applied in the designed PHC, which has a servo-vale that changes the damping constant when the mass is modified. The control ensures the desired requirements of 10*dB* maximum gain and 0.056*Hz* cutoff frequency and its response from ocean wave presents an attenuation between 88% and 95%, however the required volume of 99*m*^3^ is the main issue for practical application.

Semi-active controls in function of displacement and mass are applied in a compensator with accumulator volume of 49*m*^3^. The sprung mass has a large variation during offshore drilling. Thus, adapting control law for mass change is key aspect to ensure the success of the semi-active control to achieve the require specifications of attenuation. Control based on continuous balance and mass adaptation is applied and compared with control based on continuous skyhook and mass adaptation, with a clear advantage for the skyhook strategy due to its smaller accumulator volume of 18*m*^3^, which represents a 64% volume reduction compared to the balance control.

Overall the SAHC skyhook strategy has the best results for real applications: required heave compensation, small accumulator volume(18 *m*^3^),reasonable energy consumption and the ability to deal with large masses changes (from 150 tones to 350 tones).

This kind of controllers present jerk and chattering (see for more information [33]). These problems can be mitigated by the insertion of a low-pass filter on the control output. Future works should also include measurement noise and actuator model that are not yet addressed on the control design.

## Nomenclature

*A*: Cylinder area, *m*^2^
*b*: Viscous damping coefficient, *Ns*/*m*
*b*_1_: Virtual Viscous damping constant used in skyhook control, *Ns*/*m*
*b*_2_: Virtual Viscous damping constant used in skyhook control, *Ns*/*m*
*b*_*pas*_: Damping used by servo-valve in semi-active control in function of sprung mass, *Ns*/*m*
*b*_*sky*_: Damping used by servo-valve in skyhook semi-active control, *Ns*/*m*
*b*_*bal*_: Damping used by servo-valve in balance semi-active control in function of sprung mass, *Ns*/*m*
*b*_*Tr*−*Bal*_: Damping used by servo-valve in the traditional balance semi-active control, *Ns*/*m*
*b*_*d*_: Desired damping in balance control, *Ns*/*m*
*c*: Viscous friction coefficient of cylinder, *Ns*/*m*
*C*_*qR*_: Hydraulic conductivity, *m*^5^/(*Ns*)
*D*: Controller transfer function of suspension system
*g*: Gravity, *m*/*s*^2^
*ι*: Length of pipeline, *m*
*i*: Complex number
*l*: Dimensionless factor that relates the cutoff frequency with the natural frequency
*K*: Bulk modulus, *Pa*
*k*: Accumulator stiffness, *N*/*m*
*k*_2_: Desired accumulator stiffness in balance control, *N*/*m*
*M*: Sprung mass, *kg*
*M*_*last*_: The previous state of Sprung mass, *kg*
*P*_*atm*_: Atmospheric pressure, *Pa*
*p*_*E*_: Cylinder oil pressure, *Pa*
*p*_*E*0_: Static pressure of accumulator gas, *Pa*
*p*_*G*_: Accumulator gas pressure, *Pa*
*p*_*G*0_: Static pressure of cylinder oil, *Pa*
*P*_*max*_: Maximum pressure of PHC, *Pa*
*r*: Polytropic coefficient
*s*: Laplace domain variable, *rad*/*s*
*s*_*b*_: Minimum frequency which the impedance simplification is valid, *rad*/*s*
*t*: time, *s*
*T*: Transmittance
*U*: Force developed by suspension system, *N*
*V*_*E*_: Oil volume, *m*^3^
*V*_*G*0_: Gas volume, *m*^3^
*V*_*G*0−*last*_: The previous state of Volume, *m*^3^
*x*_*p*_: Heave displacement of sprung mass, *m*
*x*_*h*_: Heave displacement of ship, *m*
Δ*p*_*E*_: Small variation of cylinder oil pressure about equilibrium point, *Pa*
Δ*p*_*G*_: Small variation of accumulator gas pressure about equilibrium point, *Pa*
*ω*_*c*_: Cutoff frequency, *rad*/*s*
*ω*_*n*_: Natural frequency of heave compensator, *rad*/*s*.
*ζ*: Damping coefficient of heave compensator.

## Supporting information

S1 Appendix Transmittance error due to neglect bulk modulus. This error is analyzed in order to determine whether the bulk modulus can be neglected.
(PDF)

S2 Appendix Controllers robustness. The robustness of each controller is shown with the frequency response when there is variation in the sprung mass and the damping of servo-valve.
(PDF)

S3 Appendix Stability analysis of skhook and balance control strategies. (PDF)
