# Supplementary material for: Passive and semi-active heave compensator: Project design methodology and control strategies
Source: PLoS One. 2017 Aug 16;12(8):e0183140. doi: 10.1371/journal.pone.0183140 (PMC5558971; doi:10.1371/journal.pone.0183140)
Supplement: S1 Appendix — This error is analyzed in order to determine whether the bulk modulus can be neglected. (PDF) [file pone.0183140.s001.pdf]

Appendix S1: Transmittance error due to neglect bulk modulus.

The equivalent electrical diagram of the compensator is given by Fig S1.1, where Fig S1.1A and Fig S1.1B are with and without bulk modulus respectively. The main components are the resistances (R_1 and R_2), the capacitances (C_1 and C_2), the inductance of sprung mass (L) and the speeds ($\dot{x}_p$ and $\dot{x}_h$), which are analogous to currents.

where figs A and B

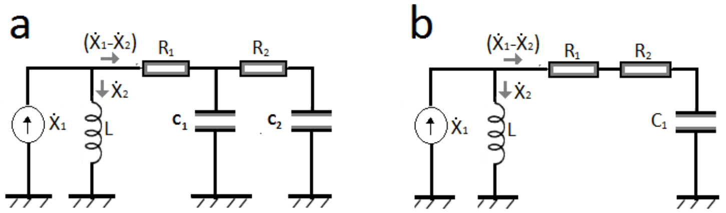

Fig S1.1. Equivalent electrical diagram. (a) With bulk modulus, (b) Without bulk modulus.

The resistance R_1 corresponds with the viscous damping coefficient of cylinder c, while the resistance R_2 is related to the system damping. Moreover, capacitance C_2 represents the pneumatic capacity of gas accumulator. The unique difference between the diagrams is the absence of capacitance C_1 in Fig S1.1B, because C_1 represents bulk modulus. The relation between the electrical parameters and mechanical parameters are:

$$\begin{aligned} L &= M \\ R_1 &= c \\ R_2 &= \frac{A^2}{C_{qR}} \\ C_1 &= \frac{V_E}{KA^2} \\ C_2 &= \frac{V_{G0}}{rP_{G0}A^2} \end{aligned}$$

The circuits of Fig S1.1 are equivalent to the circuit of Fig S1.2. Therefore, there is an equivalent impedance for each case, with and without C_1. Fig S1.2 shows currents (which are equivalent to velocities) through each element.

The voltage applied on inductance (L) and on equivalent impedance (I_{eq}) is the same. This voltage represents the force-deflection of the suspension, which is described by:

$$L\frac{d\dot{x}_h(t)}{dt} = I_{eq}(\dot{x}_p(t) - \dot{x}_h(t)) \tag{S1.2}$$

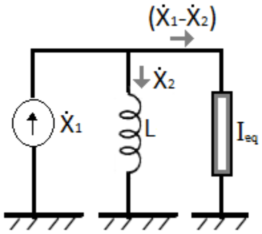

Fig S1.2. Circuit equivalent of PHC

Equivalent circuit transmittance is obtained by applying the Laplace transform:

$$T(s) = \frac{x_h(s)}{x_p(s)} = \frac{I_{eq}(s)}{Ms + I_{eq}(s)} \tag{S1.3}$$

The same transmittance equation is obtained in [1], which uses a control approach for the design of passive suspension. Such approach facilitates analysis of the one-degree-of-freedom system [2]. The transmittance requires an equivalent impedance for both cases.

The impedance expression without bulk modulus $I_{without-K}(s)$ is easy to calculate, because it is a series circuit $R_1 + R_2 + C_2$ with impedance:

$$I_{without-K}(s) = \frac{sC_2(R_1 + R_2) + 1}{sC_2} \tag{S1.4}$$

The impedance with bulk modulus $I_{with-K}(s)$ is obtained by considering $R_1 + (C_1//(R_2 + C_2))$. In this case, the impedance is:

$$I_{with-K}(s) = \frac{R_1 sC_1(sC_2R_2 + 1) + (sC_2(R_1 + R_2) + 1)}{sC_1(sC_2R_2 + 1) + sC_2} \tag{S1.5}$$

If $\|(C_1C_2R_2s + C_1)\| << C_2$, $I_{without-K} \approx I_{with-K}$. For practical applications $\|(C_1C_2R_2s + C_1)\| < nC_2$, an acceptable approximation is obtained by $n = 0.03$. Isolating the variable s of this simplification, the frequency in this point is s_b, which represents the maximum frequency value where the simplification is valid. Eq. (S1.6) presents the s_b calculation.

$$s_b = \frac{1}{R_2}\sqrt{\frac{n^2}{C_1^2} - \frac{1}{C_2^2}} \tag{S1.6}$$

The results show that the bulk modulus has a minor impact on the performance of the PHC in frequencies of interest, subsection Bulk modulus effect shows the frequency response with and without bulk modulus.

Nomenclature.

Complementary nomenclature of the main document.

C_1	-	Cylinder oil capacitance, m/N
C_2	-	Accumulator gas capacitance, m/N
Δ_I	-	Impedance variation when is not considered the bulk modulus
Δ	-	Impedance variation divided by impedance with bulk modulus
I_{eq}	-	Suspension system impedance
I_{with-K}	-	Impedance with bulk modulus
$I_{without-K}$	-	Impedance without bulk modulus
L	-	Inductance of sprung mass
R_1	-	Cylinder resistance, Ns/m
R_2	-	Valve resistance, Ns/m

References

[1] Moreau X, Ramus C, Oustaloup,A. . Fractional differentiation in passive vibration control. Nonlinear Dynamics. Jul, 2002. doi: 10.1023/A:1016518118007

[2] Rizzo A, Moreau X, Oustaloup A, Hernette V. A New CRONE Suspension: More Compact and More Efficient: Part 1Problematics and Specifications. In ASME 2009 International Design Engineering Technical Conferences and Computers and Information in Engineering Conference. American Society of Mechanical Engineers. doi:10.1115/DETC2009-86783

[3] Theron N, Els P. Controllable wheeled vehicle suspension research. PRETORIA UNIV , SOUTH AFRICA; 2002.
