# Supplementary material for: Passive and semi-active heave compensator: Project design methodology and control strategies
Source: PLoS One. 2017 Aug 16;12(8):e0183140. doi: 10.1371/journal.pone.0183140 (PMC5558971; doi:10.1371/journal.pone.0183140)
Supplement: S2 Appendix — The robustness of each controller is shown with the frequency response when there is variation in the sprung mass and the damping of servo-valve. (PDF) [file pone.0183140.s002.pdf]

Appendix S2: Controllers Robustness.

Robustness is the ability of the control to be insensitive to component variations. This appendix characterizes the controllers' robustness when there is parameters variation. The figures present the frequency response without control, with control without parameter variation and with control with parameter variation. The suspended mass M and damping of the servo valve $b_{control}$ are the changing parameters, which are used by the controllers. The parameter variation is 10% around the real value ($0.9M$, $1.1M$, $0.9b_{control}$ and $1.1b_{control}$), the frequency response of the control is shown for the four controllers, semi-active control of maximum gain $10dB$, semi-active control of maximum gain $3dB$, skyhook control and balance control.

The frequency response of semi-active control $10dB$ with parameters variation has a maximum gain variation of 8%. The gain variation around the frequency $0.17Hz$ is 2.5%, this frequency value belongs to attenuation band. The cutoff frequency is almost the same as when there is no parameter variation (see Fig S2.1). The frequency response variation is less than 8% with a parameter variation of 10%.

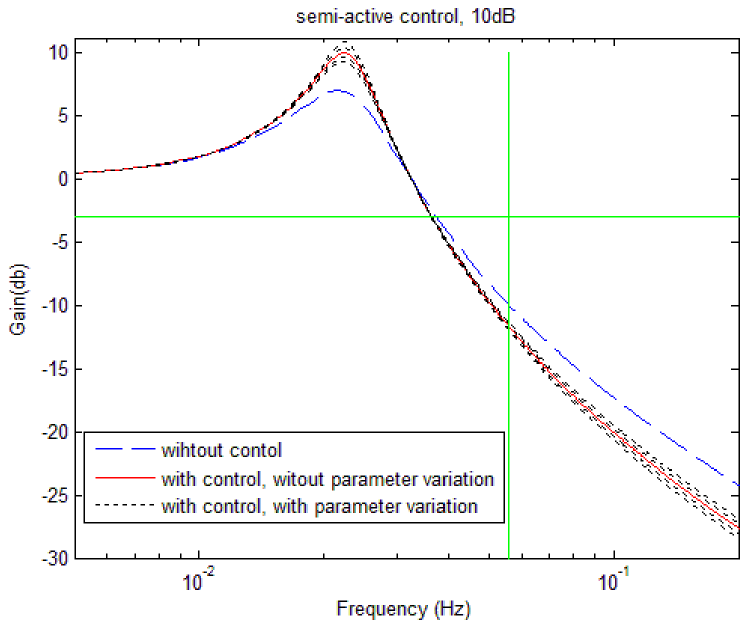

Fig S2.1. Robustness of semiactive control of maximum gain 10dB.

Fig S2.2 shows the frequency response of semi-active control $3dB$ with parameters variation. The maximum gain variation is 13%. The gain variation around the frequency $0.17Hz$ is 4%. The cutoff frequency is almost the same as when there is no parameter variation. Even with parameter variation the

response has better attenuation in transition band that the system without control.

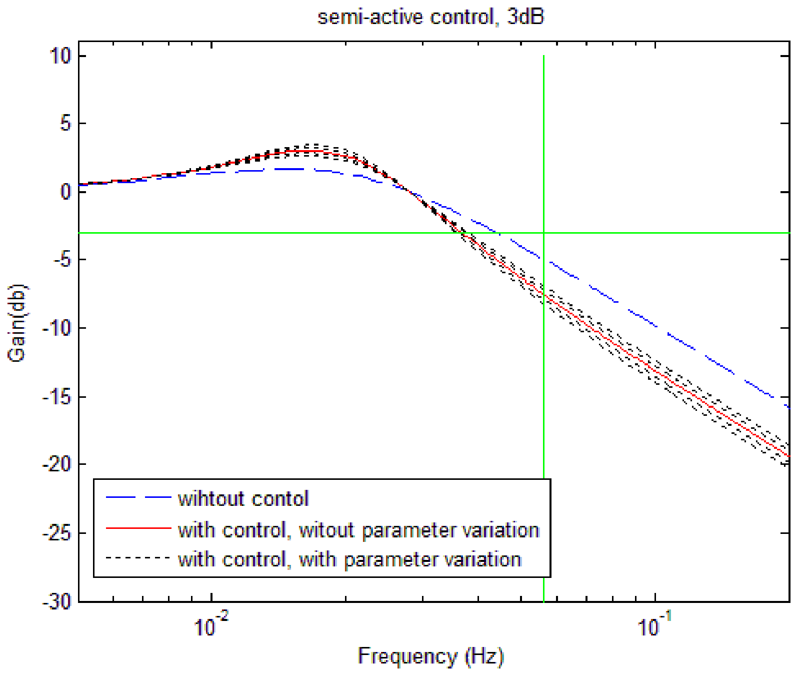

Fig S2.2. Robustness of semiactive control of maximum gain 3dB.

Fig S2.3 shows the frequency responses of balance control. It presents high variation of maximum gain $\pm 1dB$ S2.3, representing $\pm 25\%$ of the desired value. This control has the maximum variation of maximum gain. However, the cutoff frequency $0.056Hz$ is respected and the variation in transition band is acceptable.

The skyhook control has a better performance in terms of changes in parameters, because the frequency response with variation is almost the same as the frequency response obtained without parameters variation (see Fig S2.4). The skyhook control has a robustness for variation of 10% of sprung mass and damping.

The controllers are not fully robust, because there are changes in frequency responses, specially in its maximum gain, which presents a variation between 25% and 0%, balance and skyhook, respectively. However, the controllers respected the cutoff frequency $0.056Hz$ and the attenuation in transition band is acceptable. For these reasons the frequency response is acceptable in terms of parameters changes. The case of skyhook response has a better robustness, because with the parameters variations, the response is almost the same.

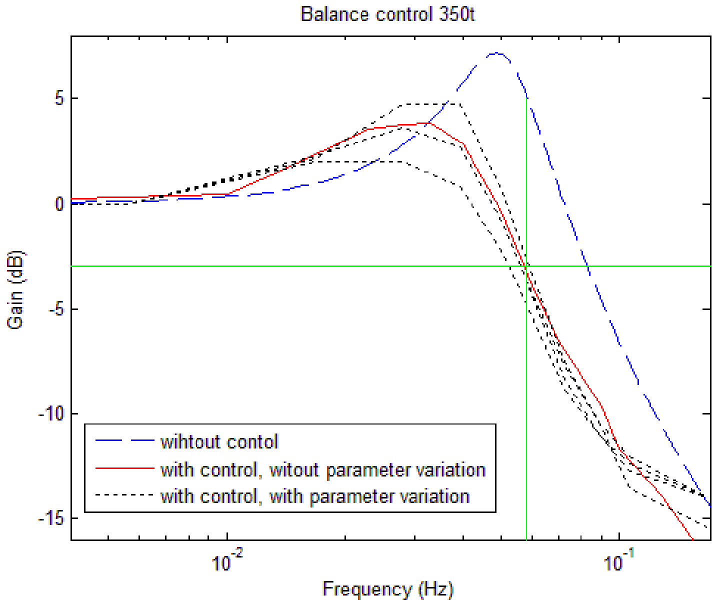

Fig S2.3. Robustness of balance control.

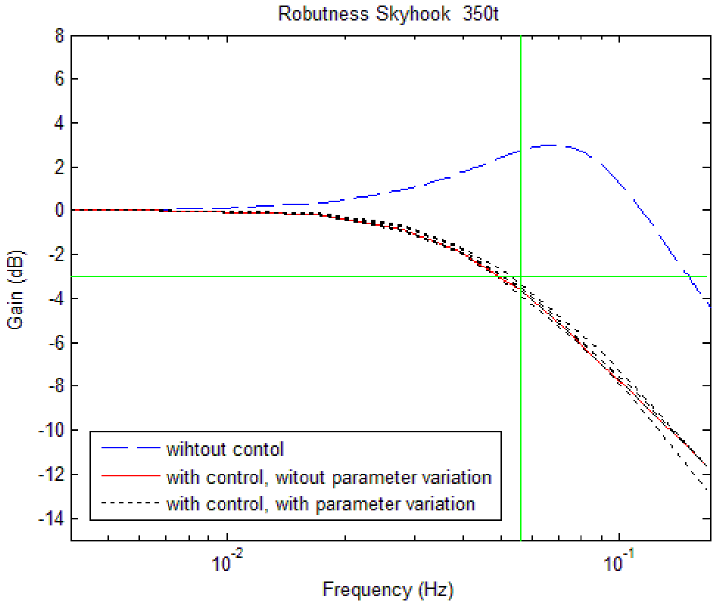

Fig S2.4. Robustness of control balance.
