# Supplementary material for: Passive and semi-active heave compensator: Project design methodology and control strategies
Source: PLoS One. 2017 Aug 16;12(8):e0183140. doi: 10.1371/journal.pone.0183140 (PMC5558971; doi:10.1371/journal.pone.0183140)
Supplement: S3 Appendix — (PDF) [file pone.0183140.s003.pdf]

# S3 Appendix: stability analysis of skyhook and balance control strategies.

This appendix presents the stability analysis for the proposed semi-active controllers. Considering the system dynamics Eq. (13), which takes into account the control input saturation

$$\ddot{x}_p = k\left(x_h - x_p\right) + \left(b_{control} + c\right)\left(\dot{x}_h - \dot{x}_p\right) \tag{S3.1}$$

$b_{control}$ is the damping from the semi-active control command. The semi-active control laws of the skyhook and balance are defined in Eq. (5) and Eq. (3), respectively.

$$b_{sky}(\dot{x}_p, M) = b_1(M) + b_2(M)\frac{\dot{x}_p}{\dot{x}_p - \dot{x}_h} \tag{S3.2}$$

$$b_{bal}(\dot{x}_p, M) = b_d(M) + (k_d(M) - k(M))\frac{x_p - x_h}{\dot{x}_p - \dot{x}_h} \tag{S3.3}$$

The control command $b_{control}$ is obtained using the following saturation due to physical limitations and $b(\dot{x}_p, M)$ from one of the control laws presented in Eq. (2) and Eq. (3).

$$b_{control} = \begin{Bmatrix} b_{max}, & b(\dot{x}_p, M) \geq b_{max} \\ b_{control}, & b_{max} > b(\dot{x}_p, M) > 0 \\ 0, & 0 \geq b(\dot{x}_p, M) \end{Bmatrix} \tag{S3.4}$$

The presented stability proof only considers the boundary values of $b_{control}$. Thus both control strategies have the same proof as well as same boundary values.

Let $w = kx_h + (c + b_{control})\dot{x}_h$, then

$$\ddot{x}_p = -\frac{k}{m}x_p - \frac{b_{control} + c}{m}\dot{x}_p + \frac{1}{m}w. \tag{S3.5}$$

The Lyapunov candidate function is defined as

$$V = \mathbf{X^T P X} \tag{S3.6}$$

where $X$ is the state variables and $P$ is a positive matrix

$$\mathbf{X} = \begin{Bmatrix} x_p \\ \dot{x}_p \end{Bmatrix} \quad \mathbf{P} = \begin{bmatrix} \frac{k}{m}(c + b_{max} + m) & 1 \\ 1 & (c + b_{max} + m) \end{bmatrix} \tag{S3.7}$$

For the matrix $P$ be positive definite, the determinant of the matrix $P$ must be greater than zero, $det(P) > 0$. The determinant of $P$ is

$$det(P) = k\left(2b_{max} + 2c + m + \frac{(b_{max}+c)^2}{m}\right) - 1 > 0 \tag{S3.8}$$

The heave compensator has $k >> 1$, $b_{max} >> 1$, $c >> 1$. Thus, the determinant of $P$ is always bigger than 0 and $P$ is a positive definite matrix. Now, the derivative of $V$ is obtained

$$\begin{aligned}\dot{V} = \quad & +2\tfrac{1}{m}w\left(x_p + (c+b_{max}+m)\dot{x}_p\right) - 2\tfrac{k}{m}x_p^2 - 2x_p\dot{x}_p\tfrac{(c+b_{control})}{m} \\ & -2\dot{x}_p^2\left((c+b_{max}+m)\tfrac{(c+b_{control})}{m} - 1\right)\end{aligned} \tag{S3.9}$$

Defining the matrix L

$$\mathbf{L} = \begin{bmatrix} 2\frac{k}{m} & \frac{c+b_{control}}{m} \\ \frac{c+b_{control}}{m} & 2\left[\frac{(c+b_{control})(b_{max}+c+m)}{m} - 1\right]\end{bmatrix} \tag{S3.10}$$

The determinant of L has the below expression, which is always positive, because $c > 1$ and $b_{max} > b_{control} > 0$. Thus $L$ is positive definite.

$$det(\mathbf{L}) = \begin{matrix} (c+b_{control}-1)\frac{4k}{m} \\ +\frac{c+b_{control}}{m^2}\left(4k(c+b_{max}) - (c+b_{control})\right)\end{matrix} > 0 \tag{S3.11}$$

The positive matrix $L$ is used to rewrite the Eq. (**??**)

$$\dot{V} = -\mathbf{X^TLX} + 2\frac{1}{m}w\left(x_p + (c+b_{max}+m)\dot{x}_p\right) \tag{S3.12}$$

When $x_h$, $\dot{x}_h$ and $b_{control}$ are bounded, $w$ is also bounded, $\|w\| \leq w_0$. We write $x_p + (c+b_{max}+m)\dot{x}_p$ as $YX$, where $Y = [1, (c+b_{max}+m)]$ and use the inequality $YX \leq \|X\|\,\|Y\|$. The norm of $Y$ is constant. Using the last two inequalities we obtain:

$$\dot{V} \leq -\mathbf{X^TLX} + \frac{2}{m}\|Y\|\,\|X\|\,w_0 \tag{S3.13}$$

The variables $\lambda_{min}$ and $\lambda_{max}$ denote the smallest and the largest eigenvalues of a matrix, respectively.

$$\dot{V} \leq -\lambda_{min}(L)\,\|X\|^2 + \frac{2}{m}\|Y\|\,w_0\,\|X\| \tag{S3.14}$$

A part of $-\lambda_{min}(L)\,\|X\|^2$ dominates $\frac{2}{m}\|Y\|\,w_0\,\|X\|$ for large $\|X\|$. The inequality is rewritten as

$$\dot{V} \leq -(1-\theta)\lambda_{min}(L)\left\|X\right\|^2 - \theta\lambda_{min}(L)\left\|X\right\|^2 + \frac{2}{m}\left\|Y\right\| w_0 \left\|X\right\| \tag{S3.15}$$

where $0 < \theta < 1$. Then,

$$\dot{V} \leq -(1-\theta)\lambda_{min}(L)\left\|X\right\|^2, \ \forall \ \left\|X\right\| \geq \frac{2}{m}\left\|Y\right\| w_0 \frac{1}{\theta\lambda_{min}(L)} \tag{S3.16}$$

We conclude that the solutions are globally uniformly ultimately bounded $B$. The theorem 4.18 of [**?**] is applied to determine the ultimate bound:

$$B = \frac{2}{m}\left\|Y\right\| w_0 \frac{1}{\theta\lambda_{min}(L)}\sqrt{\frac{\lambda_{max}(P)}{\lambda_{min}(P)}} \tag{S3.17}$$

# References


[1] Khalil H. Nonlinear Systems. 2002.
